# Artificial Intelligence in Lung Cancer: A Narrative Review of Recent Advances in Diagnosis, Biomarker Discovery, and Drug Development

**DOI:** 10.3390/pharmaceutics18020201

**Published:** 2026-02-03

**Authors:** Srikanth Basety, Renuka Gudepu, Aditya Velidandi

**Affiliations:** 1 Tris Pharma, Monmouth Junction, NJ 08852, USA; srikanth_basety@yahoo.com; 2 Department of Microbiology, Pingle Government College for Women (A), Warangal 506370, India; renumanduva@gmail.com; 3 Department of Biotechnology, Vaagdevi Degree and P.G. College, Warangal 506001, India

**Keywords:** artificial intelligence, biomarker discovery, deep learning, drug development, lung cancer diagnosis, machine learning, personalized medicine, radiomics

## Abstract

This review highlights the rapidly evolving role of artificial intelligence (AI) in transforming lung cancer care, with a specific focus on its integrated applications across diagnosis, biomarker discovery, and drug development. The novelty of this work lies in its holistic examination of how AI bridges these traditionally separate domains, from radiology and pathology to genomics and clinical trials, to create a more cohesive and personalized oncology pipeline. We detail how AI algorithms significantly enhance early detection by improving the accuracy and efficiency of pulmonary nodule characterization on computed tomography scans and enable precise cancer subtyping via computational pathology. In biomarker discovery, AI-driven analysis of radiomic features and genomic data facilitates the non-invasive prediction of tumor genotype, PD-L1 expression, and immunotherapy response, moving beyond invasive tissue biopsies. Furthermore, AI is accelerating the drug development lifecycle by identifying novel therapeutic targets and optimizing patient selection for clinical trials. The review also explores AI’s critical role in personalizing treatment regimens, including predicting outcomes for radiotherapy and immunotherapy, thereby tailoring therapy to individual patient profiles. We critically address the challenges of clinical translation, including model interpretability, data standardization, and ethical considerations, which are pivotal for real-world implementation. Finally, we contend that the future of lung cancer management hinges on robust, multi-institutional validation of AI tools and the development of trustworthy, explainable systems.

## 1. Introduction

### 1.1. Lung Cancer: Statistics

Lung cancer remains a significant global health challenge, being the leading cause of cancer-related deaths worldwide (Table 1). In 2021, the global prevalence of lung cancer was approximately 3.25 million cases, with an age-standardized prevalence rate of 37.3 per 100,000 people [1]. The incidence of lung cancer in 2022 was estimated at 2.48 million new cases, with an age-standardized incidence rate of 23.6 per 100,000 [2]. In 2020, there were 2.21 million new cases, with the highest incidence rates observed in Europe and Asia [3]. The incidence rates are notably higher in regions with high human development index (HDI), with high HDI countries having age-standardized incidence rates approximately 8.5 times those of low-HDI countries [2].

Lung cancer accounted for approximately 1.8 million deaths in 2020, making it the leading cause of cancer mortality globally [3]. The age-standardized mortality rate was 18.0 per 100,000 in 2020, with significant regional variations [4]. In 2022, the age-standardized mortality rate was 16.8 per 100,000, with higher rates in high HDI countries compared to low-HDI countries [2]. The mortality-to-incidence ratio, a proxy for 5-year survival rates, was 0.71 globally, indicating a high mortality rate relative to incidence [5]. Regions such as Eastern Asia and Eastern Europe have some of the highest mortality rates, with Eastern Asia alone accounting for nearly half of the global lung cancer deaths [5].

High-income regions, such as the high-income Asia Pacific, had the highest age-standardized prevalence rate, while East Asia had the highest age-standardized incidence rate and age-standardized mortality rate [1]. In 2020, Hungary had the highest age-standardized mortality rate at 42.4 per 100,000, while Nigeria had the lowest at 0.86 per 100,000 [4]. In 2018, Asia accounted for 58.5% of global lung cancer cases, followed by Europe with 22.4% [6]. The incidence and mortality rates were significantly higher in countries with higher tobacco smoking prevalence and HDI [4]. Regions with higher HDI scores generally have higher lung cancer mortality rates, although the mortality-to-incidence ratio is lower, suggesting better survival outcomes [7]. Lung cancer incidence and mortality rates are approximately twice as high in men compared to women [3]. In China, the age-standardized incidence rate and age-standardized mortality rate for male lung cancer patients were significantly higher than those for females [2].

From 1990 to 2019, lung cancer deaths increased by 91.75%, reaching over 2 million deaths in 2019 [8]. By 2050, the global burden of lung cancer is expected to increase, with projections of 3.8 million new cases and 3.2 million deaths annually [4]. In China, projections for 2050 estimate 1.8 million new cases and 1.4 million deaths, highlighting the need for effective prevention strategies [2]. The increase in lung cancer cases is largely attributed to population aging and persistent risk factors such as smoking and air pollution [9]. The United States is expected to see approximately 330,000 new cases and 200,000 deaths by 2050, with a notable gender disparity in incidence and mortality rates [2]. In Europe and North America, lung cancer incidence among males is projected to decline, while rates among females are expected to increase or plateau, reflecting changing smoking patterns [10].

### 1.2. Lung Cancer: Challenge and the Promise of Artificial Intelligence

Lung cancer poses significant global health challenges, representing the second most common cause of cancer-related fatalities worldwide [11]. A major contributing factor to its high mortality rate is the inherent difficulty of early detection, often due to a lack of discernible symptoms in its initial stages [11]. Beyond early detection, challenges persist in achieving precise diagnoses, ensuring effective treatment, and overcoming resistance to therapies [11,12]. For example, differentiating between adenocarcinoma in situ and early-stage invasive adenocarcinoma is complicated by the intrinsic structural heterogeneity of exosomes [13]. Furthermore, biomarkers like carcinoembryonic antigen, while indicative, are not specific to lung cancer, manifesting in other cancers such as gastric, pancreatic, colorectal, and breast cancer, which complicates their diagnostic utility [14]. Treatment resistance, particularly to targeted therapy and immunotherapy in non-small cell lung cancer (NSCLC), remains a significant clinical obstacle [12]. Additionally, systemic disparities in access to preventive services, affecting ethnically and socioeconomically marginalized groups, represent an ongoing societal challenge [15].

Amidst these profound challenges, artificial intelligence (AI) has emerged as a promising technology with the potential to transform lung cancer management across screening, diagnosis, prognosis, and treatment selection [16,17,18]. Its promise lies in augmenting professional capabilities and providing innovative solutions to long-standing problems [15,19].

In the realm of screening and early detection, AI holds substantial promise. Research has consistently evaluated AI’s role and future potential in lung cancer screening, particularly highlighting its efficiency in the classification of pulmonary nodules [16]. The process involves a series of steps, including image acquisition, pre-processing, segmentation of regions of interest, feature calculation, feature engineering, and the construction of classification models [20]. AI methods, with a particular emphasis on radiomics and deep learning (DL), are central to the detection and diagnosis of pulmonary nodules in lung imaging [20]. When deployed as a standalone algorithm for tumor identification on chest radiographs, AI classification has demonstrated performance equivalent to that of an average radiologist [21]. Machine learning (ML), a subset of AI, has proven highly effective in predicting various types of cancer, including lung cancer, by learning from training data [17]. Advanced DL models are increasingly applied for early lung cancer detection, with ongoing exploration into model architecture, data preprocessing techniques, and performance metrics [22]. A diverse array of ML and DL methods, such as convolutional neural networks (CNNs), random forest, ensemble extreme boosting (XGBoost), support vector machine, AdaBoost classification model, long short-term memory networks, and incremental multiple resolution residual network models, are being compared for their accuracy in lung cancer prediction [11]. Recent robust AI approaches integrating multiple computed tomography (CT) datasets have achieved impressive diagnostic results, with an accuracy of 99.38%, precision, specificity, and area under the curve of 100%, sensitivity of 98.76%, and an F1-score of 99.37% [23].

The utility of AI extends significantly into cancer prognosis, prediction, and personalized treatment selection. AI algorithms leveraging radiomics features have been evaluated for their efficacy in predicting epidermal growth factor receptor (EGFR) mutation status in NSCLC patients, offering crucial insights for targeted therapies [24]. AI approaches facilitate the exploitation of high-dimension oncological data, which is vital for research and development in precision immuno-oncology and the discovery of predictive biomarkers [25]. For example, an AI-driven stemness-related gene signature has been constructed to decipher prognosis and immunotherapy response in lung adenocarcinoma [26]. This involves using CytoTRACE analysis of single-cell RNA sequencing data to identify genes associated with stemness in lung adenocarcinoma epithelial cells [26]. The integration of AI and ML algorithms in these areas aims to provide a more critical and informed approach to cancer prediction, prognosis, and treatment selection [17].

To further enhance diagnostic capabilities, multi-modal AI approaches are being explored, which integrate radiological data with clinical records and genetic markers to create more personalized diagnostic tools [22]. Acknowledging the challenge of opacity, where professionals grapple with the lack of transparency in AI tools for critical medical judgments [19], there is a growing emphasis on explainable AI to provide clarity and trustworthiness in predictions [11,23]. This ensures that AI not only performs effectively but also offers understandable insights into its decision-making process [11]. Moreover, AI methods are viewed as essential tools to improve research methods and bolster the ability to enhance outcomes, particularly in addressing disparities in access to preventive services for marginalized groups [15]. While the widespread utilization of AI in cancer screening programs across regions like ASEAN is still being explored [27], the integration of robust AI models with diverse datasets holds significant promise for achieving equity and improving outcomes across the entire lung cancer diagnostic continuum [15,23]. Ongoing research into mechanisms of resistance to targeted therapy and immunotherapy in NSCLC will also benefit from AI-driven insights to develop new therapeutic approaches [12].

In summary, lung cancer management is fraught with complex challenges, from the necessity of early detection and precise diagnosis to overcoming treatment resistance and ensuring equitable access to care. AI, encompassing ML and DL, offers a powerful promise to address these hurdles (Table 2). Its applications span improved screening through efficient nodule classification, enhanced diagnostic accuracy comparable to human experts, and the prediction of treatment response and prognosis through biomarker discovery and gene signatures. Despite challenges such as AI opacity that require careful consideration, continuous advancements in AI methodologies, including multi-modal data integration and explainable AI, suggest a transformative future. This future envisions AI playing a pivotal role in achieving earlier detection, personalized treatment, and ultimately, significantly improving patient outcomes in the global fight against lung cancer.

## 2. A Primer on Artificial Intelligence Methodologies in Medicine

The integration of AI methodologies into medicine has garnered significant attention across various domains, driven by the potential to enhance diagnostic accuracy, treatment efficacy, and healthcare delivery. This section offers insights from the recent literature, emphasizing core AI approaches such as ML, DL, natural language processing (NLP), reinforcement learning (RL), and their applications within medical contexts.

ML stands out as a foundational approach that underpins many AI advancements in healthcare. As described by Smith et al. [28], ML involves the use of computer algorithms that learn from data to identify patterns and make predictions. Its capacity to improve decision-making processes has been demonstrated across multiple medical fields, including emergency medicine, where ML models assist in rapid diagnosis and triage. The ability of ML to handle complex, high-dimensional datasets makes it particularly suitable for clinical applications, as highlighted by Miller et al. [29], who discussed its role in clinical trials and healthcare research. The application of ML in clinical trials exemplifies how algorithms can optimize patient selection, predict outcomes, and streamline the development of new therapies.

DL, a subset of ML characterized by neural networks with multiple layers, has been instrumental in advancing medical imaging and diagnostics. For instance, Ahmed et al. [30] emphasized DL’s transformative potential in pancreatic imaging, where it leverages large datasets to improve image analysis, segmentation, and feature extraction. Similarly, in musculoskeletal applications, end-to-end design of reconstruction and segmentation techniques has been a focus, as noted by Tong et al. [31], demonstrated how DL models enhance image quality and diagnostic precision. The ability of DL to automatically learn hierarchical features from raw data has led to significant improvements in radiology, pathology, and other imaging modalities.

CNNs have become the cornerstone of medical image analysis due to their exceptional ability to recognize complex patterns within visual data. The advent of DL approaches, especially CNNs, has significantly surpassed traditional image recognition techniques, leading to improved performance in radiology and other imaging modalities [32]. CNNs are designed to process volumetric and 2D images, enabling detailed feature extraction that is crucial for accurate diagnosis. For instance, in musculoskeletal applications, CNNs assist in reducing the reporting burden on radiologists by automating the interpretation of imaging data [31]. Similarly, in radiology, CNNs facilitate the detection and classification of abnormalities, thereby transforming the diagnostic workflow [33]. The architecture of CNNs allows for hierarchical feature learning, which is particularly advantageous in medical imaging where subtle differences can be clinically significant [32].

NLP has also gained prominence in medical AI applications, especially in processing unstructured clinical notes and textual data. NLP techniques help extract meaningful information from vast amounts of free-text data, which traditionally posed challenges for manual review. According to Melnyk et al. [34], NLP enables complex analysis of large datasets, allowing clinicians and researchers to extract meaningful insights from textual information. This capability supports tasks such as clinical documentation, decision support, and literature mining, thereby improving the efficiency and accuracy of medical workflows. Ye et al. [35] demonstrated the utility of NLP combined with ML to predict mortality in critically ill diabetic patients by leveraging clinical notes and Unified Medical Language System resources. This approach underscores NLP’s capacity to interpret complex clinical narratives and integrate them with structured data for predictive modeling. Furthermore, NLP techniques are instrumental in reducing the reporting burden on clinicians and radiologists by automating the extraction of relevant information from reports and electronic health records (EHRs) [33]. The ability of NLP to facilitate multimodal data fusion, such as combining textual and imaging data, enhances the comprehensiveness of clinical assessments [36].

Radiomics, a rapidly evolving field, involves the extraction of high-dimensional quantitative features from medical images to characterize tissue heterogeneity and disease phenotypes. The application of AI, particularly DL and CNNs, has propelled radiomics into a new era of precision medicine. AI-driven radiomics models can analyze large datasets to identify subtle imaging features that correlate with clinical outcomes, thereby aiding in prognosis and treatment planning [37]. The integration of DL techniques with radiomics allows for the development of more robust and automated feature extraction processes, reducing reliance on manual segmentation and subjective interpretation [38]. This synergy between AI and radiomics has the potential to uncover novel biomarkers and improve the predictive accuracy of imaging-based models [37].

RL, a paradigm where algorithms learn optimal actions through trial-and-error interactions with the environment, has shown promise in handling medical uncertainty and decision-making processes. As detailed by Jayaraman et al. [39], RL approaches are being explored for diagnosis and treatment planning, where they can adapt strategies based on patient responses and evolving clinical data. The potential of RL to develop visual-action AI agents for medical diagnosis and treatment underscores its future role in personalized medicine and adaptive clinical interventions.

In addition to these core methodologies, generative AI techniques, including generative adversarial networks, are emerging as powerful tools for data augmentation, image synthesis, and predictive modeling. Melnyk et al. [34] highlighted how generative models facilitate complex analyses, especially when data scarcity is an issue. These approaches are particularly relevant in medical imaging, where they can generate realistic synthetic data to train robust models, thereby overcoming limitations posed by limited annotated datasets.

The application of AI in medical education and regulation also reflects the broad scope of these methodologies. As discussed by Waldman et al. [40], integrating AI into medical curricula is essential for preparing future clinicians to utilize these tools effectively. Furthermore, ref. [41] underscored the importance of developing regulatory frameworks to ensure the safe and ethical deployment of AI systems in healthcare, emphasizing that the potential benefits are contingent upon responsible governance. Despite the rapid advancements, challenges remain in standardizing AI methodologies and establishing gold standards for conduct and reporting, as noted by Badrulhisham et al. [42]. The lack of uniform guidelines hampers reproducibility and validation of AI models, which is critical for clinical adoption. Addressing these issues requires ongoing efforts to develop comprehensive frameworks that ensure transparency, robustness, and ethical compliance.

In summary, the landscape of AI methodologies in medicine is characterized by a diverse array of approaches, each contributing uniquely to healthcare innovation. ML and DL form the backbone of many applications, from imaging to clinical decision support, while NLP and RL expand the scope to unstructured data analysis and adaptive decision-making (Table 3). As these technologies continue to evolve, their integration into clinical practice promises to revolutionize medicine, provided that ethical, regulatory, and methodological challenges are adequately addressed. The ongoing research and development in this field, as reflected across the literature, underscore a future where AI-driven tools become integral to medical science and patient care. While each AI methodology offers unique strengths, their clinical translation is often hampered by modality-specific limitations. Table 4 provides a comparative overview of key AI strategies—radiomics, genomics, multimodal integration, DL, liquid biopsy, and digital pathology—highlighting their principal challenges and proposing context-aware solutions. This critical appraisal serves as a foundation for understanding the trade-offs and strategic selections discussed in subsequent application-focused sections.

## 3. Data Landscapes for Artificial Intelligence in Lung Cancer

**Figure 1:**
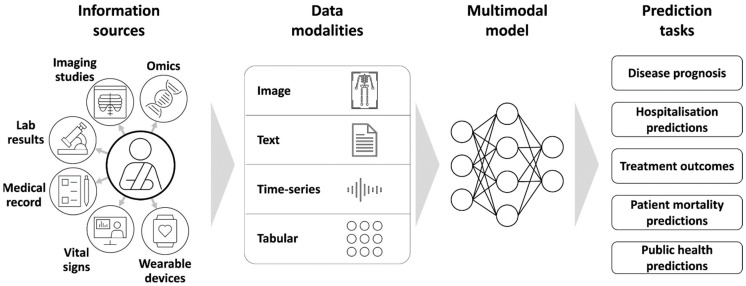
Clinical data types and predictive outcomes. Reproduced from [36].

The landscape of data utilized for AI applications in lung cancer diagnosis, prognosis, and treatment is remarkably diverse, encompassing medical imaging, genomic data, EHRs, and real-world data (RWD) (Figure 1). Each data modality offers unique insights, and their integration is increasingly recognized as pivotal for advancing precision oncology.

Medical imaging, such as radiology data, forms a cornerstone of lung cancer diagnostics. According to recent studies, the separation of data sources across hospital, academic, and commercial entities responsible for imaging, histopathology, and genomic sequencing presents significant challenges but also opportunities for comprehensive data integration [44]. Automated RWD integration techniques have been developed to bridge these gaps, enabling more cohesive datasets that enhance cancer outcome predictions. Such integration facilitates the application of AI algorithms to imaging data, improving detection accuracy and enabling early diagnosis [44].

Genomic data, including DNA sequencing and RNA expression profiles, constitute another critical data landscape. The advent of clinico-genomic databases, such as the Flatiron Health-Foundation Medicine United States based database, has provided a wealth of real-world genomic information linked with clinical data [45]. These datasets allowed for the characterization of mutation-treatment effects, which are essential for understanding tumor heterogeneity and tailoring personalized therapies. Moreover, the use of de-identified EHRs combined with genomic data has been instrumental in uncovering mutation patterns associated with treatment responses [45]. European perspectives also highlight the importance of real-world genomic data in precision oncology, emphasizing the need for standardized data collection and integration [46].

EHRs serve as a rich repository of patient-specific clinical information, including diagnostic reports, procedural notes, and unstructured clinical narratives. Transforming this unstructured data into structured, discrete elements is a critical step for AI applications. Recent advancements have demonstrated the use of NLP techniques to convert EHR notes into analyzable data, thereby facilitating predictive modeling and decision support [47]. AI-driven analysis of EHRs has shown promise in unveiling patient trajectories, predicting disease progression, and identifying potential biomarkers for lung cancer [48]. Furthermore, integrating EHR data with other modalities, such as imaging and genomics, enhances the depth and accuracy of predictive models [49].

RWD, encompassing diverse sources like EHRs, claims data, and patient registries, has gained prominence for its role in reflecting actual clinical practice outside controlled trial settings. The utilization of RWD in AI models has led to significant insights into treatment effectiveness, patient outcomes, and disease heterogeneity in lung cancer. For instance, DL algorithms trained on RWD have demonstrated improved predictive capabilities for patient prognosis [48]. Despite its potential, RWD remains underutilized in certain contexts, partly due to challenges in data standardization and integration across different sources [43].

The convergence of these data landscapes is exemplified by efforts to develop comprehensive patient models that incorporate imaging, genomic, and clinical data. For example, the development of deep patient graph convolutional networks (DeePaN) integrates EHRs and genomic data to predict treatment responses and disease progression [50]. Such integrative approaches leverage the strengths of each modality, providing a more holistic view of the patient’s disease state and enabling more precise interventions. Furthermore, the application of foundation models—large-scale AI models trained on extensive datasets—coupled with chromatin QTL and other genomic annotations, exemplifies the cutting-edge integration of multi-omics and clinical data [51]. These models aim to uncover complex biological mechanisms underlying lung cancer and identify novel biomarkers, thereby accelerating translational research.

In summary, the various data landscapes—medical imaging, genomic data, EHRs, and RWD—are increasingly being integrated to enhance AI-driven lung cancer research and clinical care. Each modality contributes unique insights: imaging provides morphological details, genomics offers molecular characterization, EHRs supply comprehensive clinical histories, and RWD reflects real-world treatment patterns and outcomes (Table 5). The ongoing efforts to automate data integration, standardize data formats, and develop sophisticated AI models are crucial for translating these rich datasets into actionable clinical insights, ultimately improving patient outcomes in lung cancer.

## 4. Artificial Intelligence in Diagnosis and Early Detection

The application of AI has fundamentally reshaped the initial phases of the lung cancer care continuum, with its most profound impact felt in diagnosis and early detection. By leveraging advanced algorithms on diverse data types—from radiological images to pathological slides—AI systems enhance the accuracy, speed, and objectivity of identifying and characterizing lung cancer. This section critically examines these transformative applications, from automated nodule detection on CT scans to computational pathology and the integration of multi-modal data for diagnostic refinement.

### 4.1. Artificial Intelligence for Pulmonary Nodule Detection and Characterization on Computed Tomography

**Figure 2:**
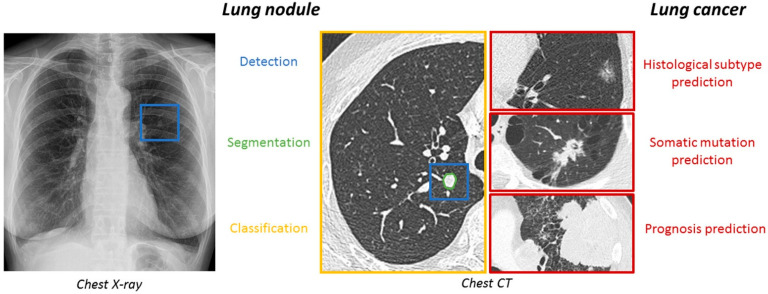
Presentation of DL applications for lung nodule and cancer analysis in chest imaging. Reproduced from [53].

A comprehensive review of recent advancements in AI for pulmonary nodule detection and characterization on CT highlighted significant progress driven by DL techniques, particularly CNNs. These aim to enhance early lung cancer detection, improve diagnostic accuracy, and facilitate clinical decision-making, although several challenges remain before routine clinical integration. The DL has demonstrated promising capabilities in pulmonary nodule detection, segmentation, and classification. Tandon et al. [52] emphasized that CNNs have shown notable success in these areas, with the potential to predict lung cancer and classify nodules effectively. Similarly, Margerie-Mellon and Chassagnon [53] noted that the proliferation of CNN-based applications has led to performances reaching or surpassing radiologists’ accuracy in automated detection tasks. These models leverage large imaging datasets to learn complex features associated with benign and malignant nodules, facilitating more precise diagnoses (Figure 2).

The performance of AI systems has been evaluated across various imaging modalities and settings. Chamberlin et al. [54] reported on an AI CNN prototype (AI-RAD Companion) that automatically detects pulmonary nodules and quantifies coronary artery calcium volume on low-dose CT scans, comparing its accuracy to expert radiologists. Their findings suggested that AI can achieve high accuracy in nodule detection, which is crucial for lung cancer screening programs. Similarly, Jungblut et al. [55] evaluated an AI-based computer aided design system in photon-counting detector CT at different low-dose levels, demonstrating superior image quality and comparable performance to traditional imaging techniques.

The influence of advanced CT technologies on AI performance has also been explored. Jungblut et al. [55] found that photon-counting detector CT provided better subjective image quality than energy-integrating detector CT, with AI systems performing effectively at various dose levels. Yao et al. [56] further investigated the impact of DL image reconstruction algorithms on ultra-low-dose CT, showing that DL image reconstruction improved detection rates and image quality metrics such as signal-to-noise ratio and contrast-to-noise ratio. These studies underscore the importance of high-quality imaging for optimal AI performance.

**Figure 3:**
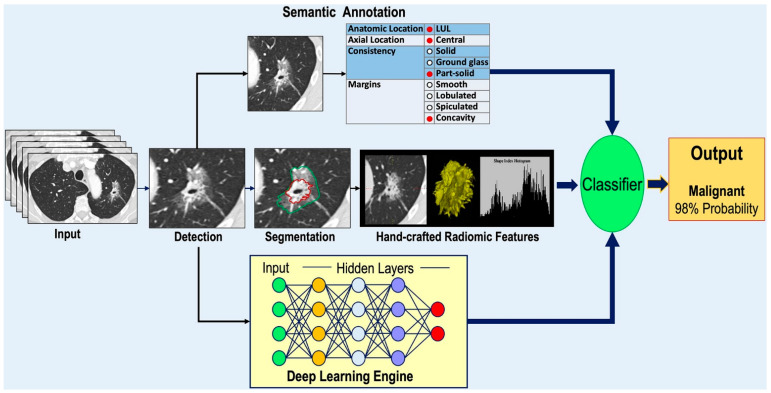
Diagram outlines the primary methods for classifying indeterminate pulmonary nodules and characterizing early lung cancer. Following the detection of a lung nodule on a CT scan, it can be processed through three main pathways: Annotation with semantic terms, segmentation for radiomic feature extraction, or direct analysis by a DL model. The results from each of these approaches are then fed into a classifier, which generates an output such as the probability of malignancy or a prediction of the tumor’s histology and genetic profile. Reproduced from [59].

In terms of detection accuracy, several studies have demonstrated the potential of AI to distinguish benign from malignant nodules. Du et al. [57] conducted a retrospective analysis comparing AI-based diagnosis with physician assessments and postoperative pathology, revealing that AI can provide reliable diagnostic support. Despite these promising results, challenges persist. Pedrosa et al. [58] highlighted that integrating AI into routine clinical practice requires addressing issues such as data heterogeneity, model robustness, and validation across diverse populations. The Lung Nodule Database Challenge exemplified efforts to standardize datasets and evaluate AI systems holistically, aiming to improve generalizability and clinical applicability [58]. Similarly, Prosper et al. [59] reviewed the application of radiomic features and ML models in early lung cancer characterization, emphasizing the need for overcoming methodological obstacles like feature selection and data annotation to facilitate clinical translation (Figure 3).

The issue of false positives remains a significant concern. Schreuder et al. [60] discussed the high false positive rates associated with AI in lung cancer screening, which can lead to unnecessary follow-ups and patient anxiety. They advocate for AI-human collaboration, where AI assists radiologists rather than replacing them, to optimize diagnostic accuracy and cost-effectiveness. This collaborative approach is echoed by Chassagnon et al. [61], who noted that AI adoption in thoracic imaging is progressing toward clinical integration, provided that performance levels are validated and standardized. Furthermore, the reliability and consistency of AI models are critical for clinical deployment. Vasilev et al. [62] evaluated the robustness of commercially available radiological AI systems across datasets acquired before and during the COVID-19 pandemic, finding minimal performance variation and supporting the potential for widespread application. In addition to detection, AI has shown promise in characterizing pulmonary nodules. Prosper et al. [59] reviewed how ML models utilizing CT-derived radiomic features can aid in early lung cancer characterization, although they acknowledge the technical challenges posed by heterogeneous imaging parameters and the necessity for well-annotated datasets. Similarly, Nagase et al. [63] developed an AI-driven 3D CT prediction model for preoperative assessment of visceral pleural invasion, demonstrating AI’s expanding role in comprehensive lung cancer staging.

In summary, the recent literature underscored the significant strides made in applying AI, particularly DL and CNNs, for pulmonary nodule detection and characterization on CT. These systems have demonstrated high accuracy, robustness, and potential to augment radiological workflows. Nonetheless, challenges such as false positives, data heterogeneity, validation, and clinical integration remain. Ongoing efforts focusing on standardization, quality assurance, and collaborative human-AI approaches are essential to realize the full potential of AI in routine lung cancer screening and diagnosis.

### 4.2. Lung Cancer Subtyping and Grading via Computational Pathology

The advent of computational pathology has significantly advanced the precision and objectivity of lung cancer subtyping and grading. This section highlights current developments, emphasizing AI-driven methodologies, validation studies of histological grading systems, and the integration of DL techniques for improved prognostication and classification.

A foundational aspect of computational pathology in lung cancer involves the validation and application of histological grading systems. The IASLC grading system for invasive pulmonary adenocarcinoma has been a focal point in recent studies. Deng et al. [64] conducted a retrospective analysis of 950 Chinese patients, validating the prognostic utility of the IASLC grading system. Their findings demonstrated that the grading system effectively stratifies patients based on prognosis, underscoring its clinical relevance. Similarly, Rokutan-Kurata et al. [65] evaluated the same grading system in a Japanese cohort, confirming its prognostic significance. Both studies highlight the importance of standardized histological grading in lung adenocarcinoma, facilitating consistent prognostic assessments across diverse populations.

**Figure 4:**
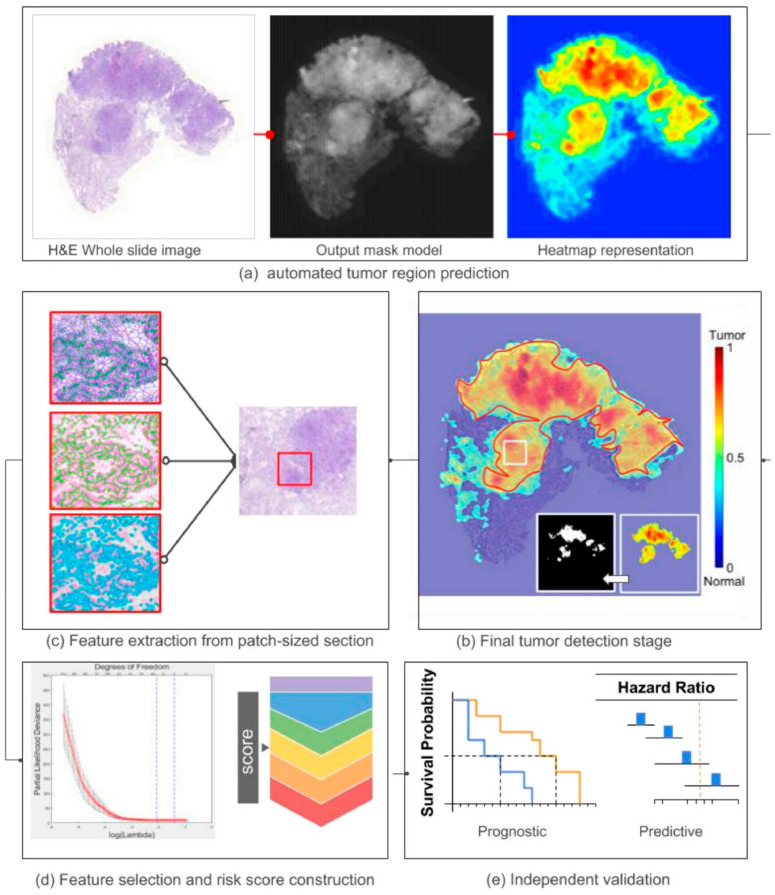
Flowchart of the overall methodology: (**a**) automated identification of the tumor region; (**b**) extraction of image tiles from within the defined tumor area; (**c**) computation of cellular features from nuclei and their surrounding regions; (**d**) selection of key features and subsequent calculation of the computational pathology risk score; (**e**) assessment of the computational pathology risk score’s value for predicting patient prognosis and treatment response. Reproduced from [67].

Despite these advances, the integration of AI and computational methods offers promising avenues for enhancing lung cancer subtyping and grading. Laak [66] discussed the current landscape of AI in histopathology, emphasizing that while numerous AI applications have been developed, few studies have incorporated external validation with large, independent cohorts. This gap underscores the necessity for prospective validation to establish clinical utility. The potential of AI to automate and refine grading processes is further exemplified by Wang et al. [67], who developed a computational image signature that predicts prognosis and the benefit of adjuvant chemotherapy in early-stage NSCLC (Figure 4). Their work demonstrated how computational pathology can inform treatment decisions, moving beyond traditional histological assessment.

The DL techniques, particularly those leveraging whole-slide images (WSIs), have been instrumental in advancing lung cancer subtyping. Lu et al. [68] introduced a weakly supervised DL method, CLAM, which efficiently processes WSIs by focusing on diagnostically relevant subregions. This approach reduces the need for extensive annotations and enhances interpretability. Similarly, Chen et al. [69] proposed the Hierarchical Image Pyramid Transformer, which models the hierarchical structure of WSIs through self-supervised learning. Their results showed superior performance in cancer subtyping and survival prediction, emphasizing the importance of hierarchical and high-resolution representations in pathology. Attention mechanisms and multiple instance learning frameworks have also been pivotal. Zhao et al. [70] further contributed by developing RLogist, a deep RL approach for rapid WSI observation, which accelerates the diagnostic process. These methods collectively underscored the potential of attention-based and RL models to improve both the accuracy and efficiency of lung cancer grading.

**Figure 5:**
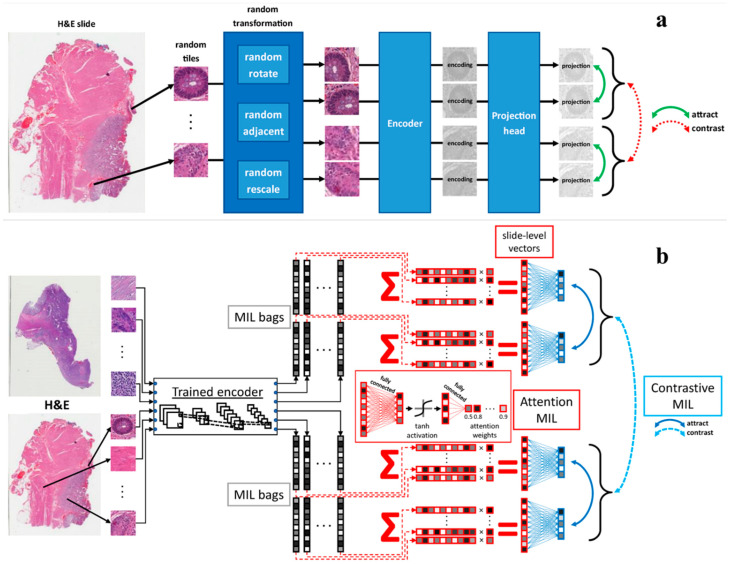
(**a**) The SimCLR framework learns generalized image tile representations by making them invariant to augmentations like rotation, scaling, and adjacent tile selection. Each tile is transformed twice, and the model is trained to identify these transformed pairs as similar while distinguishing them from all other tiles. (**b**) The tile encodings from (**a**) are used to create multiple ‘bags’ per WSI. An attention-based multiple instance learning model aggregates each bag into a slide-level representation. These slide-level representations are then refined through contrastive learning, where bags from the same slide are treated as positive pairs. Reproduced from [71].

Unsupervised and self-supervised learning paradigms are gaining traction for their ability to leverage unlabeled data. Tavolara et al. [71] presented a contrastive multiple instance learning framework that learns slide-level representations without labels, which could be particularly useful given the scarcity of annotated datasets (Figure 5). Vaidya et al. [72] addressed demographic biases in computational models, revealing that self-supervised vision foundation models can reduce performance disparities across demographic groups, thus promoting equitable diagnostic tools. The challenge of interpretability and robustness remains central. Xie et al. [73] demonstrated the utility of 3D pathology with DL assisted gland analysis, which offers a nondestructive and potentially more accurate assessment of tissue architecture. Similarly, Kassab et al. [74] proposed formalin-fixation and paraffin-embedding++, an image translation method that enhances tissue image quality, thereby improving the reliability of computational analyses.

**Figure 6:**
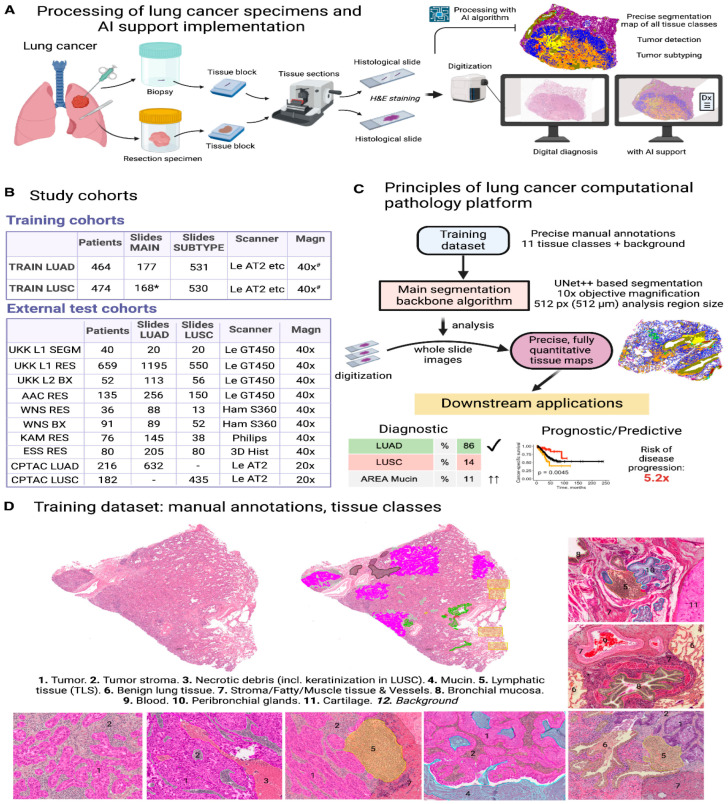
Development of computational platform for NSCLC. (**A**) Specimen processing and digital pathology, (**B**) study cohorts and annotation, (**C**) main segmentation algorithm, and (**D**) manual annotation methodology. Reproduced from [75].

In terms of clinical translation, next-generation platforms are being developed. Kludt et al. [75] created a comprehensive computational pathology platform for NSCLC (Figure 6), trained on high-quality annotated datasets, which can serve as a foundation for multiple downstream applications. Li et al. [76] challenged the conventional fine-tuning paradigm, suggesting that task-agnostic representations from foundation models can effectively adapt to WSI analysis, simplifying the deployment pipeline. Shao et al. [77] further confirmed the robustness of pretrained multiple instance learning models, showing that transfer learning significantly boosts performance even across domain shifts. Finally, the integration of multimodal data and retrieval systems is emerging as a frontier. Wang et al. [78] introduced PathSearch, a framework that aligns visual and linguistic features for efficient WSI retrieval, which could facilitate more precise subtyping and grading by enabling rapid comparison with annotated reference images.

In summary, the landscape of computational pathology for lung cancer subtyping and grading is rapidly evolving, driven by validation of histological grading systems, innovative DL architectures, and efforts to improve interpretability and clinical applicability. While challenges such as external validation, demographic bias, and integration into clinical workflows persist, the collective research underscores a promising trajectory toward more accurate, scalable, and equitable lung cancer diagnostics. Continued development and validation of AI models, especially those leveraging large, diverse datasets and unsupervised learning, are essential for translating these technological advances into routine clinical practice.

### 4.3. Integration of Multi-Modal Data for Diagnostic Refinement

**Figure 7:**
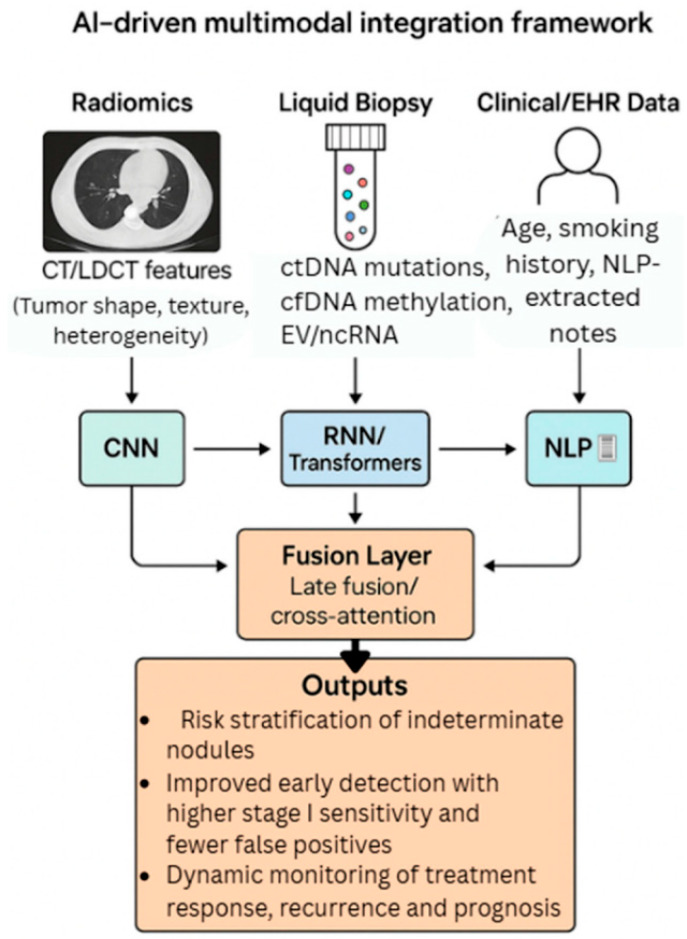
AI-driven multimodal integration framework for lung cancer detection and monitoring. Reproduced from [79].

The integration of multi-modal data using AI has emerged as a transformative approach in refining lung cancer diagnosis, offering enhanced accuracy and personalized treatment strategies (Figure 7). The recent literature underscored the significance of combining diverse data sources—such as imaging, genomic, histopathological, and clinical information—to overcome the limitations of traditional diagnostic methods and to facilitate early detection, precise classification, and prognostic assessment.

**Figure 8:**
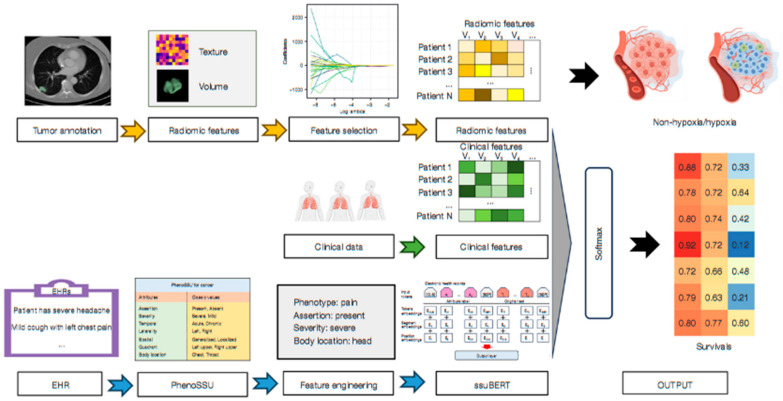
The structure of the multi-modal AI model. Reproduced from [82].

Kim et al. [80] exemplified this trend by proposing a DL ensemble method that exploits multi-modal patient data to predict recurrence in NSCLC patients. Their approach highlighted the potential of integrating various data types—possibly including imaging, clinical records, and molecular profiles—to improve predictive accuracy. This aligned with the broader consensus that multi-modal data fusion can capture the complex biological and clinical heterogeneity inherent in lung cancer, thereby enabling more reliable prognostic models. Ladbury et al. [81] emphasized the role of AI in early diagnosis and tailored therapy, advocating for models that utilize multi-modal data to support multidisciplinary teams in clinical decision-making. They argued that AI-driven models can synthesize information from multiple sources in real-time, thus facilitating timely and precise interventions. This perspective is reinforced by Zhou et al. [82], who developed an AI model for predicting hypoxia status and prognosis in NSCLC through the integration of multi-modal datasets, including radiomic features from CT scans, genomic data, and clinical parameters (Figure 8). Their study demonstrated that combining radiomic features with genomic and clinical data enhances the predictive power for critical tumor characteristics, such as hypoxia, which is associated with treatment resistance and poor prognosis.

The application of advanced DL architectures further exemplified the potential of multi-modal integration. Kumar et al. [83] assessed the effectiveness of combining magnetic resonance imaging biomarkers with various ML models—such as CNN, KNN, VGG16, and RNN—to improve lung cancer detection. Their modular approach underscored the importance of leveraging different AI techniques to process and fuse heterogeneous data types, ultimately aiming for more accurate and robust diagnostic systems. Similarly, Mgbole [84] reviewed the use of multi-modal imaging, genomics, and clinical data in early-stage cancer detection, emphasizing that ML models can effectively analyze complex datasets to identify early disease markers. Radiomics, derived from advanced imaging modalities like positron emission tomography (PET)/CT, also plays a crucial role in multi-modal data integration. Safarian et al. [85] reviewed the expanding application of [18F]FDG PET/CT radiomics combined with AI to improve diagnostic accuracy, staging, and molecular subtype identification in lung cancer. Their findings suggested that radiomics features, when integrated with AI algorithms, can enhance the detection of tumor heterogeneity and molecular markers, which are vital for personalized therapy planning. Histopathological data, when combined with genomic and imaging information, further enriches the diagnostic landscape. Abdullakutty et al. [86] explored explainable AI approaches that integrate histopathology images with other data modalities to improve breast cancer diagnosis, a methodology that can be translated to lung cancer. Their review highlighted the importance of explainability in multi-modal AI models, ensuring that clinicians can interpret and trust the outputs, which is critical for clinical adoption.

Furthermore, the potential of AI to assist in thoracic surgery and treatment planning is explored by Cusumano et al. [87], who advocated for interdisciplinary collaboration to harness AI’s capabilities safely and effectively. Their insights suggested that multi-modal data integration can support surgical decision-making, tumor resection planning, and post-operative management, thereby improving overall patient outcomes. Recent advances in NLP models, such as Meta LLaMa 3.1, demonstrated the expanding scope of AI in thoracic diagnostics, although their performance in lung cancer-specific tasks remains variable [88]. Nonetheless, these models hold promise for processing unstructured clinical notes and integrating them with imaging and molecular data, further enriching multi-modal diagnostic frameworks.

In summary, the current literature consistently underscored the transformative potential of multi-modal data integration facilitated by AI in lung cancer diagnosis. The convergence of imaging, genomic, histopathological, and clinical data through sophisticated AI models enhances diagnostic accuracy, enables early detection, and supports personalized treatment strategies. While challenges such as data heterogeneity, interpretability, and validation remain, ongoing research and technological advancements continue to push the boundaries of what is achievable. The integration of multi-modal data not only refines diagnostic precision but also paves the way for more effective, tailored therapeutic interventions, ultimately improving patient outcomes in lung cancer care.

## 5. Artificial Intelligence in Biomarker Discovery and Prognostication

Moving beyond initial detection, AI is revolutionizing the discovery of prognostic and predictive biomarkers, which are essential for personalizing lung cancer management. By decoding complex patterns within radiomic, genomic, and real-world data, AI models can non-invasively predict tumor genotype, forecast treatment response, and stratify recurrence risk. This section explores how these AI-driven approaches are uncovering novel digital and molecular signatures to guide more precise and individualized therapeutic strategies.

### 5.1. Radiomics and Deep Learning for Non-Invasive Biomarkers

The integration of radiomics and DL within AI frameworks has significantly advanced the pursuit of non-invasive biomarkers for lung cancer, offering promising avenues for early detection, prognosis, and personalized treatment strategies. The current literature underscored a multifaceted approach that leverages imaging, molecular, and liquid biopsy data to enhance diagnostic accuracy and therapeutic decision-making.

Radiomics, which involves extracting high-dimensional quantitative features from medical images such as CT, has emerged as a pivotal tool in lung cancer characterization. Dunn et al. [89] highlighted the development of automated classification systems utilizing CT-based radiomic analysis, emphasizing the importance of reliable, automated diagnostic tools. Their work demonstrated a low failure rate in tumor segmentation, which is crucial for subsequent radiomic feature extraction. Similarly, So et al. [90] presented a DL auto-segmentation pipeline designed to facilitate radiomic analysis, underscoring the role of AI in improving the precision and efficiency of tumor delineation. These advancements enable the extraction of radiomic features that can serve as non-invasive biomarkers, providing insights into tumor heterogeneity and biology without the need for invasive biopsies.

**Figure 9:**
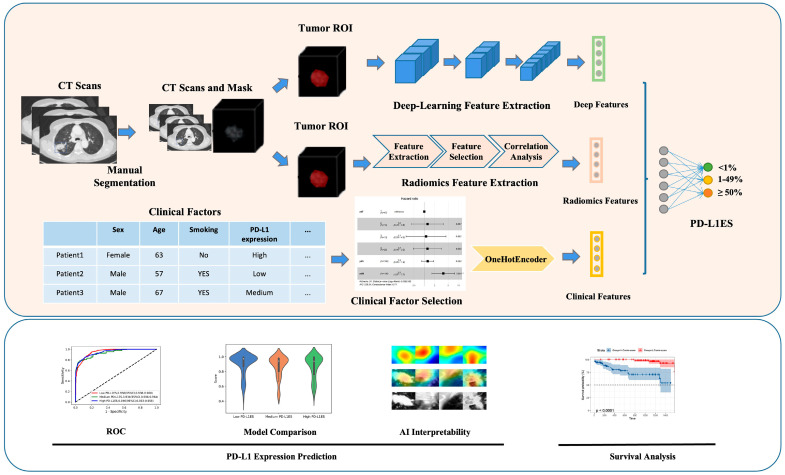
The upper section outlines the study’s overall methodology, and the lower section details the model’s analytical process. The data processing phase incorporated original CT scans with manual tumor labels, comprehensive clinical data, overall survival information, and PD-L1 expression status. During feature extraction, DL, radiomics, and clinical features were derived from the tumor regions of interest and patient records. These combined features were then used to predict PD-L1 expression and assess patient survival. Reproduced from [91].

DL, a subset of AI characterized by neural network architectures capable of learning complex patterns, has been extensively applied to enhance the predictive power of radiomics. Wang et al. [91] demonstrated that combining DL with radiomics and clinical data improves the prediction of programmed death ligand 1 (PD-L1) expression and survival in NSCLC (Figure 9). Their model, based on CT images of 1135 patients, exemplified how multi-source feature fusion can aid clinicians in rapid decision-making regarding immunotherapy and other treatments. Similarly, Mahajan et al. [92] developed a predictive imaging biomarker model for EGFR mutation status using CT images, achieving an area under curve of 0.88, which outperformed individual models. This indicated that DL models can effectively predict molecular alterations, which are critical for targeted therapies.

The predictive capabilities of AI extend beyond molecular markers to encompass immune response and treatment benefit. Lee et al. [93] introduced ‘CheckpointPx,’ a radiology AI model designed to predict benefit from immune checkpoint inhibitors using baseline CT scans. Their model demonstrated robust performance in predicting immune checkpoint inhibitors response, facilitating non-invasive treatment selection. Complementing this, Liu et al. [94] integrated longitudinal CT radiomics with DL to map the tumor immune microenvironment, deriving an Immune Evasion Score that predicts immunosuppressive niches. Such models exemplify how AI-driven radiomics can provide insights into tumor-immune interactions, which are pivotal for immunotherapy planning.

Liquid biopsies, which analyze circulating tumor DNA and other biomarkers in blood, have also been integrated with AI to develop non-invasive diagnostic tools. Bahado-Singh et al. [95] combined AI with DNA methylation analysis of circulating cell-free DNA to identify biomarkers for lung cancer detection, illustrating the potential of molecular liquid biopsies. Karimzadeh et al. [96] further explored deep generative AI models analyzing orphan non-coding RNAs from serum samples, demonstrating high accuracy in early-stage NSCLC detection. These approaches highlighted the potential of combining molecular data with AI to develop sensitive, non-invasive biomarkers that can detect lung cancer at early stages when intervention is most effective.

The application of AI extends into the realm of histopathology and genomics. Parra-Medina et al. [97] systematically reviewed DL models predicting oncogenic driver mutations from histopathological images, emphasizing the diagnostic accuracy achievable through DL. Such models can potentially replace or supplement invasive tissue biopsies, providing rapid molecular insights. Similarly, Tiwari et al. [98] reviewed current AI technologies in cancer diagnostics, including genomics and liquid biopsies, emphasizing their role in biomarker discovery and personalized medicine. Immunotherapy response prediction is another critical area where AI-driven radiomics shows promise. Wani et al. [99] introduced ‘DeepXplainer,’ an interpretable DL approach for lung cancer detection, which also offers explanations for its predictions, enhancing clinical trust. Liu et al. [94] and Lee et al. [93] further demonstrated how AI models can predict immunotherapy benefit, integrating radiomic features with immune microenvironment mapping. These models facilitated non-invasive, personalized treatment strategies, reducing reliance on invasive procedures.

The broader landscape of AI in lung cancer biomarker discovery is also characterized by systematic reviews and meta-analyses that validate the efficacy of these approaches. AlOsaimi et al. [100] and Parra-Medina et al. [97] provided evidence supporting the high diagnostic accuracy and prognostic value of AI models in identifying molecular and clinical biomarkers. Moreover, Gottardo et al. [101] discussed multi-omics approaches enabled by AI, which combine data from various biological sources to identify clinically relevant biomarker combinations, representing a paradigm shift in lung cancer diagnostics.

In summary, the convergence of radiomics and DL within AI frameworks has revolutionized the quest for non-invasive biomarkers in lung cancer. These technologies facilitate the extraction of rich, high-dimensional data from imaging and molecular sources, enabling early detection, molecular characterization, and prediction of treatment response. The integration of multi-source data—imaging, liquid biopsies, genomics, and histopathology—through AI models enhances the accuracy and clinical utility of non-invasive biomarkers. As research progresses, these approaches are poised to transform lung cancer management by enabling more precise, personalized, and less invasive diagnostic and therapeutic strategies, ultimately improving patient outcomes.

### 5.2. Artificial Intelligence in Genomic and Molecular Profiling

The integration of AI and genomic/molecular profiling has revolutionized the landscape of lung cancer diagnosis, prognosis, and personalized therapy. The recent literature underscored the role of advanced molecular techniques, particularly next-generation sequencing and liquid biopsy, in enhancing our understanding of lung cancer biology and guiding clinical decision-making.

One of the most significant advancements in this domain is the application of next-generation sequencing based profiling of circulating cell-free DNA in patients with advanced NSCLC. Abate et al. [102] highlighted that next-generation sequencing of cell-free DNA offers notable advantages, such as non-invasiveness, the ability to monitor tumor dynamics in real-time, and early detection of resistance mechanisms. These features facilitate more precise therapeutic interventions and ongoing disease management. However, the authors also acknowledged limitations, including potential false negatives due to low tumor DNA shedding and technical challenges associated with cell-free DNA analysis. Despite these pitfalls, the clinical utility of cell-free DNA profiling remains substantial, especially in guiding targeted therapies and monitoring treatment response.

Complementing cell-free DNA analysis, the use of tissue-based genomic profiling through WSI combined with DL models has emerged as a promising approach. Qu et al. [103] developed a DL framework capable of predicting genetic mutations and biological pathway activities directly from histopathological images. This approach offers a non-invasive, rapid, and cost-effective alternative to traditional molecular testing, providing insights into tumor biology that can inform targeted therapy decisions. Such AI-driven image analysis bridges the gap between morphological features and underlying molecular alterations, reinforcing the potential of integrating radiological and molecular data. Furthermore, the application of AI extends to the analysis of tumor heterogeneity and clonal relationships. Yang et al. [104] utilized comparative molecular profiling to investigate the clonal origins of multiple lung lesions, emphasizing the importance of genomic testing in understanding tumor biology. Their findings reinforced that genomic profiling can clarify whether multiple lung tumors are independent primaries or metastases, which has direct implications for treatment strategies.

In the context of early-stage disease, molecular features such as EGFR mutations have been associated with recurrence risk. Saw et al. [105] demonstrated that patients with resected EGFR positive NSCLC exhibit high recurrence rates, yet a subset remains disease-free without adjuvant therapy. This underscored the importance of individualized risk profiling, which can be enhanced through molecular data, to optimize treatment plans and avoid overtreatment.

The biological complexity of lung cancer is further illustrated by the observation that histologic phenotype correlates more strongly with transcriptomic rather than genomic features. Tang et al. [106] conducted whole-exome sequencing and microarray profiling, revealing that transcriptomic signatures are more indicative of histologic differentiation than genomic alterations. This insight suggested that integrating transcriptomic data with genomic profiling could improve tumor classification and therapeutic targeting. Emerging techniques such as spatial tissue molecular profiling using laser capture microdissection have also gained attention. Liotta et al. [107] reviewed the transition of laser capture microdissection-based proteomics from research to clinical applications, emphasizing its capacity to analyze spatial heterogeneity at the single-cell level. Combining proteomics with genomic and transcriptomic data enhances our understanding of tumor microenvironments and resistance mechanisms, paving the way for more precise interventions.

Liquid biopsy, particularly cell-free DNA analysis, has been recognized as a transformative tool in clinical practice. García-Pardo et al. [108] discussed the opportunities and challenges of integrating cell-free DNA analysis into routine care. The non-invasive nature of liquid biopsy allows for dynamic monitoring of tumor evolution, detection of actionable mutations, and early identification of resistance, which are critical for personalized therapy. Ezeife et al. [109] further evaluated the economic value of liquid biopsy, demonstrating its cost-effectiveness in the management of advanced NSCLC, especially when combined with targeted therapies.

The molecular profiling of lung tumors has also facilitated the development of targeted therapies, exemplified by the successful use of anaplastic lymphoma kinase inhibitors in patients harboring specific gene fusions. Choi et al. [110] reported a case where genomic profiling identified an anaplastic lymphoma kinase fusion, leading to targeted therapy with alectinib and significant clinical improvement. Such cases exemplify how AI-enhanced molecular diagnostics can directly translate into effective personalized treatments. AI’s role extends beyond diagnostics to understanding resistance mechanisms. Isozaki et al. [111] revealed that targeted therapies can induce mutagenic enzymes like APOBEC3A, contributing to tumor evolution and resistance. Recognizing these processes through AI-driven analysis of molecular data can inform strategies to delay or prevent resistance development. In addition to molecular profiling, AI models are increasingly used to predict tumor purity and heterogeneity from histopathological images. Gerardin et al. [112] developed AI models capable of quantifying tumor cell fractions within tissue sections, which is essential for accurate molecular analysis and treatment planning. Similarly, integrating radiomics with genomic data, as discussed by Italiano et al. [113], enhanced the prediction of genomic alterations, further supporting personalized medicine.

In summary, the convergence of AI and molecular profiling techniques has significantly advanced our understanding of lung cancer biology. These technologies enable more accurate diagnosis, risk stratification, and tailored therapeutic approaches. The literature consistently emphasized that while challenges remain—such as technical limitations and the need for validation—the potential of AI-driven genomic and molecular profiling to transform lung cancer management is profound. As research progresses, integrating multi-omics data with AI will likely lead to even more precise and effective interventions, ultimately improving patient outcomes in lung cancer care.

### 5.3. Predicting Treatment Response and Recurrence Risk

The application of AI in lung cancer management has garnered significant attention, particularly in the domains of treatment response prediction and recurrence risk assessment. Recent studies underscored the potential of AI-driven models to enhance prognostic accuracy, facilitate personalized therapy, and improve clinical outcomes.

One of the promising avenues involves the integration of metabolic profiling with AI algorithms. Liu et al. [114] developed a recurrence-associated metabolic signature specifically for patients with stage I lung adenocarcinoma. Their study highlighted the relationship between metabolic reprogramming and tumor recurrence, proposing that recurrence-associated metabolic signature can serve as a robust tool for risk stratification and therapeutic response prediction. This metabolic signature exemplifies how AI can leverage complex biochemical data to predict clinical trajectories in early-stage lung cancer.

Imaging-based AI applications have also demonstrated considerable promise. Cellina et al. [115] reviewed the role of AI in lung cancer imaging, emphasizing automated lesion detection, characterization, and outcome prediction. The combination of imaging features with clinical and laboratory data through AI models has shown potential in predicting patient outcomes and response to therapies, including immunotherapy. Similarly, Dolezal et al. [116] explored the use of deep CNNs with uncertainty estimation to predict relapse risk from histopathology slides in NSCLC patients. Their approach underscored the importance of confidence quantification in AI predictions, which could enhance clinical decision-making.

Radiomics, which involves extracting quantitative features from medical images, has been extensively studied for recurrence prediction. Libling et al. [117] provided an overview of radiomic approaches in early-stage NSCLC, supporting their utility in assessing recurrence risk. Beyond imaging, molecular biomarkers and circulating tumor DNA have been integrated into AI models to predict treatment response and recurrence. Vidal et al. [118] examined the clinical impact of presurgical circulating tumor DNA in locally advanced rectal cancer, illustrating how liquid biopsy data can inform prognosis. Although focused on rectal cancer, this approach is translatable to lung cancer, where circulating tumor DNA has been recognized as a valuable biomarker. Mansur et al. [119] reviewed AI-based biomarker research in liver cancer, emphasizing the potential of molecular markers like cell-free DNA and circulating tumor cells in early detection and prognosis, which could be adapted for lung cancer.

Immunotherapy response prediction remains a critical challenge, especially given the variable efficacy observed in different patient subsets. To et al. [120] discussed the limited response of EGFR-mutant NSCLC to anti-PD-1/PD-L1 therapies and highlighted the need for predictive models to identify likely responders. AI models, including those utilizing histopathology and molecular data, are being developed to address this gap. For instance, Hanani et al. [121] employed supervised ML, specifically the ‘CatBoost’ classifier, combined with explainability techniques like SHAP, to predict recurrence in thyroid cancer, illustrating the importance of interpretable AI models that could be adapted for lung cancer.

The broader landscape of AI in lung cancer prognosis and treatment prediction is summarized by Gandhi et al. [18], who reviewed AI’s impact on improving patient outcomes through early detection, risk stratification, and therapy response prediction. Similarly, Lococo et al. [121] emphasized the importance of integrating AI algorithms into personalized prognostic assessments, advocating for continued validation and collaboration to realize AI’s full potential in NSCLC management. In the context of early-stage lung cancer, the combination of AI with traditional clinical parameters offers a pathway toward more accurate risk stratification. Liu et al. [114] and Libling et al. [117] demonstrated that integrating metabolic signatures and radiomic features can refine recurrence predictions beyond conventional staging systems. Moreover, the use of AI in histopathology, as explored by Dolezal et al. [116], provided an additional layer of prognostic information, potentially guiding adjuvant therapy decisions.

In summary, the current literature underscored the transformative potential of AI in predicting treatment response and recurrence risk in lung cancer. From metabolic signatures and radiomics to liquid biopsies and histopathology, AI models are increasingly capable of integrating diverse data types to produce personalized prognostic assessments. While challenges remain, including model validation and clinical integration, the ongoing research indicates a promising future where AI-driven tools will become integral to lung cancer management, ultimately improving patient outcomes through tailored therapeutic strategies.

### 5.4. Artificial Intelligence in Guiding Surgical Intervention and Treatment Selection

Lung cancer remains a significant global health challenge, necessitating continuous advancements in diagnostic and therapeutic strategies to enhance patient outcomes [18]. AI has emerged as a transformative technology, demonstrating substantial progress across various aspects of lung cancer management, encompassing screening, diagnosis, and treatment [18,122]. This section highlights current research on the application of AI in guiding surgical intervention and treatment selection for lung cancer patients.

AI plays a crucial role in improving diagnostic precision and risk prediction, which are fundamental to making informed treatment decisions. For instance, models have been developed to predict the nature of pulmonary nodules in high-risk patients, assisting in the critical determination of whether to proceed with surgical intervention, as evidenced by a retrospective study involving patients who underwent lobectomy or sublobectomy [123]. Enhancing these diagnostic capabilities, AI, specifically deep CNN models, when integrated with uncertainty quantification techniques, can predict lung cancer relapse from histopathology slides following surgical resection in patients with Stage I-III NSCLC [116]. The capacity of uncertainty quantification to report confidence alongside its predictions is particularly promising for improving the clinical applicability of AI models designed to estimate risk [116].

**Figure 10:**
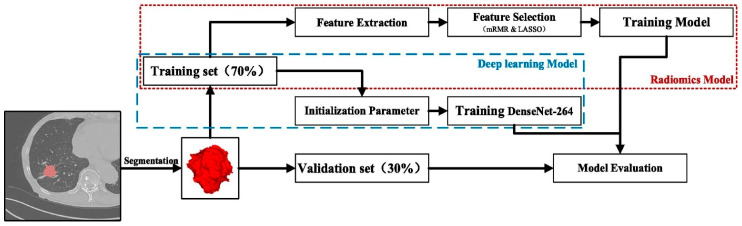
Methodology for forecasting bone metastasis in lung cancer by analyzing medical images with radiomics and DL. Reproduced from [124].

The utility of AI extends beyond initial diagnosis to predicting specific complications and treatment responses. AI-driven radiomics and DL models are under development to predict bone metastasis in lung cancer patients, thereby enhancing predictive accuracy and clinical decision-making (Figure 10) [124]. DL techniques are also being explored for their potential to predict response to therapy in complex cases. Deep pathomics, which applies DL to histological tissue slides, is being investigated to predict the response to chemoradiotherapy in Stage III NSCLC patients, addressing a significant need for personalized treatment strategies [125]. Furthermore, AI-empowered approaches show promise in differentiating lung cancer types and informing surgical choices. A clinical-radiomics nomogram, leveraging peritumoral radiomic features on CT scans, has demonstrated superior diagnostic performance for differential diagnosis in small-cell lung cancer, with its clinical utility for surgical decision-making confirmed at intermediate-risk thresholds [126]. Advances in imaging, a key domain for AI integration, are also contributing to the prediction of Spread Through Air Spaces in lung cancer, a factor with clinical significance [127]. These capabilities underscore AI’s role in providing more precise and nuanced diagnostic insights, directly influencing the decision to operate or to select a specific therapeutic pathway.

AI’s guidance is directly applicable to the selection of surgical strategies and other treatment modalities. For patients diagnosed with Stage I NSCLC, decision-making involves a critical choice among various options such as lobectomy, segmentectomy, wedge resection, stereotactic body radiation therapy, and ablation [128] s. While this systematic review did not explicitly mention AI, the framework it established for enhancing decision-making at the point of care for individual patients represents an ideal scenario for AI integration to provide personalized guidance based on comprehensive evidence [128]. Similarly, for small second primary NSCLC lesions, the comparative efficacy of wedge resection, lobectomy, and segmentectomy requires careful evaluation, a complex task that could be streamlined through AI-driven analysis [129].

Optimizing patient selection for surgery, especially in vulnerable populations, is another area where AI’s analytical capabilities prove invaluable. For elderly patients aged 75 and above, precise patient selection is crucial to improve surgical outcomes and minimize risks associated with postoperative morbidity and mortality [130]. Specific factors such as stage IIIb, pN1, pN2, and central tumor location have been identified as significant predictors of a complex postoperative period in this demographic [130]. AI can integrate such predictive risk factors with individual patient data to generate tailored recommendations, ensuring appropriate surgical candidacy. Moreover, assessing operability status and its association with post-treatment mortality in early-stage NSCLC—comparing stereotactic body radiation therapy with open or minimally invasive surgical approaches—highlights the need for sophisticated decision support systems [131]. AI’s advancements in precise medical image analysis and personalized treatment planning are considered crucial for these complex surgical interventions [122].

In summary, AI is rapidly transforming the management of lung cancer by substantially enhancing the precision and personalization of surgical intervention and treatment selection. From developing sophisticated risk prediction models for pulmonary nodules and relapse to aiding in the differential diagnosis of small-cell lung cancer with high accuracy, AI provides clinicians with invaluable tools for informed decision-making. Its capabilities extend to predicting bone metastasis and treatment response in NSCLC, thereby personalizing therapeutic strategies. The integration of AI in analyzing diverse treatment options for early-stage NSCLC and optimizing patient selection, particularly for elderly patients undergoing surgery, promises to improve outcomes and minimize complications. Furthermore, AI’s application in post-discharge care solidifies its role in supporting a holistic patient journey. As research continues to explore AI’s potential in areas like precise medical image analysis and personalized treatment planning, its impact on guiding surgical and therapeutic choices in lung cancer is poised to become even more profound, ultimately leading to improved patient outcomes.

### 5.5. Digital Biomarkers from Real-World Data and Wearables

The integration of AI into the analysis of EHRs and wearable devices data has emerged as a transformative approach in oncology, particularly in lung cancer management. This technological advancement facilitates the identification of digital biomarkers that are instrumental in assessing symptom burden, treatment tolerance, and overall survival, thereby enabling more personalized and precise care.

One of the primary roles of AI in this context is its capacity to analyze vast and complex datasets derived from EHRs and wearable devices. According to a recent review on digital health applications in oncology [132], AI-driven tools have significantly advanced clinical decision-making by extracting meaningful patterns from heterogeneous data sources. These patterns include subtle changes in patient health status that may precede clinical symptoms, thus serving as potential digital biomarkers. For lung cancer patients, such biomarkers can provide early indicators of disease progression or treatment-related adverse effects, which are crucial for timely intervention [132].

Wearable devices, equipped with sensors to monitor physiological parameters continuously, generate real-time data that AI algorithms can interpret to detect early signs of treatment-related side effects or symptom exacerbation. As noted in a study on wearable smart devices in cancer diagnosis, mobile health management systems enable the remote monitoring of patients, capturing data that was previously inaccessible outside clinical settings [133]. This continuous data stream allows AI models to identify deviations from baseline health metrics, which can be indicative of symptom burden or treatment intolerance. For example, fluctuations in activity levels, heart rate variability, or sleep patterns captured by wearables can serve as digital biomarkers linked to patient well-being and treatment response.

Furthermore, AI’s role extends to enhancing performance status assessments, which are vital in lung cancer care. A recent publication emphasized the potential of digital health tools to refine the traditional performance status evaluation, offering a more nuanced and dynamic understanding of a patient’s functional capacity [134]. Wearable devices contribute to this by providing objective, quantifiable data that can be integrated into AI models to generate a real-time performance status score. This approach not only improved the accuracy of initial assessments but also allows for ongoing monitoring, which is essential for adjusting treatment plans and predicting treatment tolerance. In addition to symptom and performance monitoring, AI facilitates the identification of prognostic biomarkers that correlate with overall survival. Biomarkers such as tumor mutation burden, immune cell profiles, and tumor microenvironment characteristics have been proposed to predict treatment response and survival outcomes [135,136]. AI algorithms can analyze complex genomic, proteomic, and clinical data to uncover novel digital biomarkers that reflect tumor biology and host response. For instance, integrating data from wearable devices with EHRs enables the development of composite biomarkers that encompass physiological, behavioral, and molecular information, providing a comprehensive picture of disease trajectory.

The application of ML, a subset of AI, has been particularly impactful in this domain. ML models can learn from large datasets to identify patterns associated with treatment tolerance and survival, which are often too subtle for traditional statistical methods [137]. These models can be trained to predict adverse events, optimize treatment regimens, and stratify patients based on their risk profiles. The potential of AI to inform lung cancer screening and treatment decisions is highlighted in recent conference reports, emphasizing its role in identifying relevant biomarkers for differential survival and treatment effects [138].

Despite these promising developments, challenges remain in translating AI-driven biomarker discovery into clinical practice. Ensuring data quality, addressing privacy concerns, and validating models across diverse populations are critical steps before widespread adoption. Nonetheless, the convergence of AI, EHR analysis, and wearable technology holds significant promise for advancing personalized lung cancer care by enabling early detection of symptom burden, improving treatment tolerance, and refining prognostic assessments.

In summary, AI plays a pivotal role in analyzing EHRs and wearable device data to identify digital biomarkers related to symptom burden, treatment tolerance, and overall survival in lung cancer. By leveraging ML algorithms to interpret complex, multimodal data, clinicians can gain deeper insights into disease progression and treatment response. This integration of digital health tools fosters a more proactive, personalized approach to lung cancer management, ultimately aiming to improve patient outcomes and quality of life.

## 6. Artificial Intelligence in Drug Development and Treatment Personalization

The transformative potential of AI extends profoundly into the realm of therapy, accelerating and refining the entire pipeline from drug discovery to clinical application. AI methodologies are being harnessed to identify novel drug targets, repurpose existing therapies, predict patient-specific responses to treatments like immunotherapy and radiotherapy, and even simulate clinical trials through digital twins. This section delves into how AI is paving the way for a new era of highly personalized, efficient, and effective therapeutic interventions in lung cancer.

### 6.1. Artificial Intelligence Accelerated Drug Discovery and Repurposing

The integration of AI into drug discovery and repurposing has emerged as a transformative approach in lung cancer research, offering promising avenues for rapid therapeutic development and personalized treatment strategies. The current literature underscored the multifaceted applications of AI, ranging from molecular target identification to high-throughput screening, and highlights the potential to overcome traditional challenges associated with lung cancer therapy.

One of the foundational aspects of AI-driven drug discovery in lung cancer involves understanding the complex molecular mechanisms underpinning tumorigenesis. For instance, Wu et al. [139] demonstrated the utility of AI-based biomedical image analysis techniques, such as the Tuna Swarm Algorithm combined with DL models, to identify and classify lung cancer from biomedical images. This approach enhanced diagnostic accuracy and facilitates early detection, which is crucial for effective treatment. Similarly, Dwivedi et al. [140] proposed an explainable AI framework that leverages DL to discover biomarkers specific to NSCLC. Their methodology employed an autoencoder to reduce feature space complexity, followed by neural network classification and biomarker identification, exemplifying how AI can streamline the biomarker discovery process for lung cancer subtypes.

**Figure 11:**
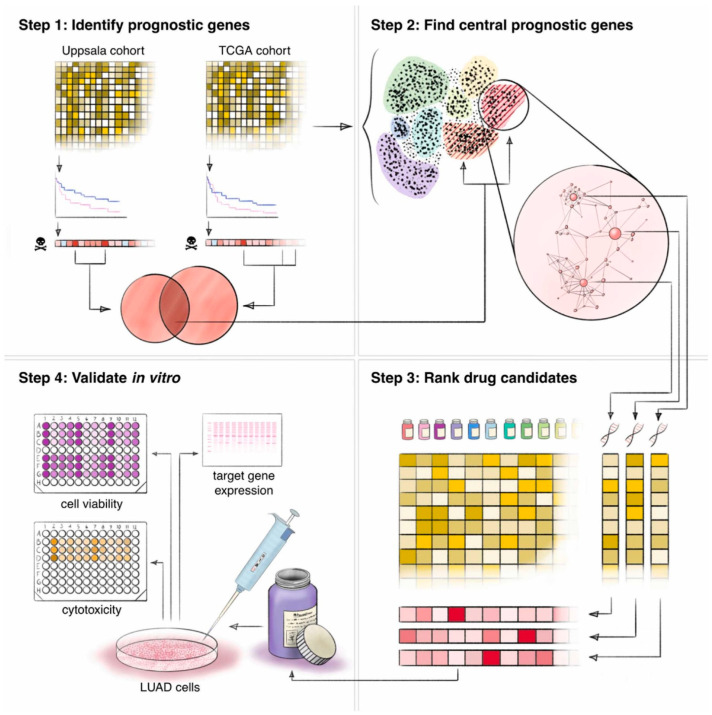
Study design for lung adenocarcinoma target identification and drug repositioning. Step 1: Prognostic gene identification. Step 2: Network and target discover. Step 3: Drug repositioning. Step 4: Experimental validation. Reproduced from [141].

Beyond diagnostics, AI has been instrumental in identifying novel therapeutic targets and repurposing existing drugs. Graves et al. [141] utilized gene co-expression network analysis combined with survival data to pinpoint hub genes driving lung adenocarcinoma, providing potential targets for drug development (Figure 11). Similarly, Ramesh et al. [142] employed high-throughput screening strategies integrated with AI to identify Food and Drug Administration approved compounds, such as Montelukast, as potential inhibitors of REarranged during Transfection, a receptor tyrosine kinase implicated in NSCLC. This exemplified how AI accelerated the repurposing process by efficiently screening vast chemical libraries against relevant molecular targets.

The application of AI extends to understanding off-target effects and toxicity profiles, which are critical for drug safety and efficacy. Lyu et al. [142] conducted proteome-wide analyses to identify off-target interactions of EGFR inhibitors, providing insights into potential toxicities and resistance mechanisms. Such studies underscored AI’s capacity to predict adverse effects and guide the optimization of drug candidates for lung cancer. In addition to molecular and target-based approaches, AI-driven network pharmacology offers a systems-level perspective. Joshi et al. [143] discussed how network pharmacology principles facilitated the construction of drug-target and disease-gene networks, moving beyond the traditional one-drug-one-target paradigm. This approach enabled the identification of multi-target drugs and combination therapies, which are particularly relevant for complex diseases like lung cancer that involve multiple dysregulated pathways.

**Figure 12:**
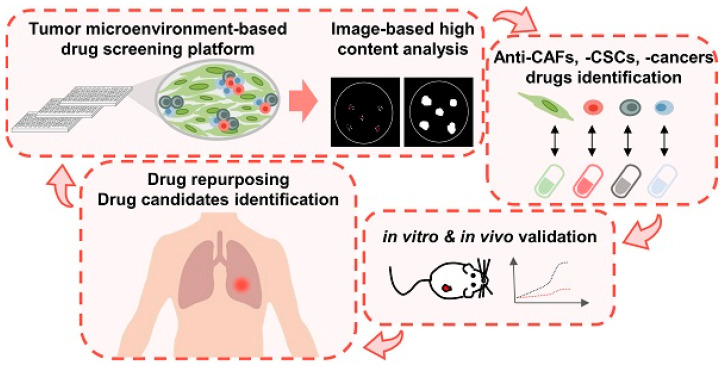
Tumor microenvironment-based screening to identify drugs that can specifically target cancer stem cells and cancer-associated fibroblasts in the tumor microenvironment. Reproduced from [145].

The integration of AI with innovative experimental models further enhances drug discovery efforts. Hwang et al. [144] highlighted the potential of organ-on-a-chip technology to evaluate drug efficacy in lung cancer models, providing a more physiologically relevant platform for testing AI-predicted drug candidates. Such models can simulate tumor microenvironments, including interactions with cancer stem cells and fibroblasts, as demonstrated by Lee et al. [145], who established a tumor microenvironment-based screening platform to identify drugs targeting cancer stem cells and associated fibroblasts (Figure 12). These studies exemplified how AI can be combined with advanced experimental systems to improve the predictive power of preclinical testing. The role of AI in identifying novel drug candidates is further exemplified by Tran et al. [146], who employed DL to discover Z29077885, an antiviral agent with potential anticancer activity targeting STK33. This demonstrated AI’s capacity to repurpose drugs from unrelated therapeutic areas, thereby expanding the repertoire of available treatments for lung cancer. Similarly, Sufyan et al. [147] reviewed the current status of AI and ML in cancer diagnosis and therapy, emphasizing their potential to accelerate drug discovery pipelines and improve personalized treatment strategies.

Despite these advances, challenges remain in translating AI-driven discoveries into clinical practice. Huang et al. [148] discussed the current applications and future perspectives of AI in lung cancer, emphasizing the need for robust validation, interpretability, and integration with clinical workflows. Moreover, the complexity of lung cancer biology necessitates comprehensive approaches that combine AI with systems biology, network pharmacology, and experimental validation.

In summary, the literature highlighted the transformative potential of AI in accelerating drug discovery and repurposing for lung cancer. From biomarker identification and target discovery to high-throughput screening and toxicity prediction, AI tools are enabling more efficient and precise therapeutic development. The integration of AI with innovative experimental models, such as organ-on-a-chip systems, further enhances the translational potential of these discoveries. As the field advances, addressing challenges related to validation, interpretability, and clinical integration will be essential to fully realize AI’s promise in improving lung cancer outcomes.

### 6.2. Predictive Models for Immunotherapy Outcomes

The landscape of lung cancer treatment has been significantly transformed by immunotherapy, which has improved outcomes across various cancer types, including lung cancer [149,150]. However, patient responses to immunotherapy are highly variable, necessitating the development of predictive models to personalize treatment strategies [150,151]. AI has emerged as a powerful tool in this endeavor, promising to enhance precision medicine in oncology.

AI’s potential in lung cancer management extends broadly, from early diagnosis and screening to personalized treatment [16,152]. AI tools can classify nodules in lung cancer screening [16] and are increasingly applied across the preoperative, intraoperative, and postoperative phases of NSCLC treatment [153]. For precision medicine, AI analysis is crucial for identifying suitable therapeutic targets and individual treatment strategies for patients with lung cancer [154]. Specifically, AI facilitates the integrated analysis of multi-omics data (such as genomics, transcriptomics, proteomics) and clinical information to discover new biomarkers and guide personalized medicine in NSCLC [150,155]. Such advances leverage large-scale clinical and imaging datasets to identify intricate patterns and predictive features that might be overlooked by human interpretation [152].

The application of AI in predicting immunotherapy outcomes for lung cancer patients is a rapidly evolving field [25,150]. These AI methods integrate diverse data types, including radiomics, pathology, genomics, transcriptomics, proteomics, and clinical data, to build comprehensive predictive models [150]. Several specific AI applications and models have been developed to predict immunotherapy responses in lung cancer. Radiomics-based models have shown promise in predicting clinical outcomes for NSCLC patients undergoing immunotherapy [156]. These models extract features from baseline and follow-up PET/CT scans (delta-radiomics) to predict durable clinical benefit, progression, response to therapy, overall survival, and progression-free survival [156]. An example is ‘CheckpointPx,’ a non-invasive radiology AI tool that uses only baseline CT scans to predict the benefit of immune checkpoint inhibitor therapy in NSCLC patients, assisting in treatment selection [93].

In pathology and image analysis, AI-powered tools are improving predictive capabilities. An AI-powered PD-L1 analyser for NSCLC has been developed to reduce interobserver variation in tumor proportion score and enhance the prediction of immunotherapy response [157]. This prototype model was built using 802 NSCLC whole-slide images [157]. Furthermore, ML analysis of pathological images has been applied to predict 1-year progression-free survival of immunotherapy in patients with extensive-stage small-cell lung cancer receiving chemoimmunotherapy [157]. The computational integration of radiology and pathology image features also holds potential for predicting treatment benefit and outcome in lung cancer, including advanced NSCLC, demonstrating improved predictive performance [158].

**Figure 13:**
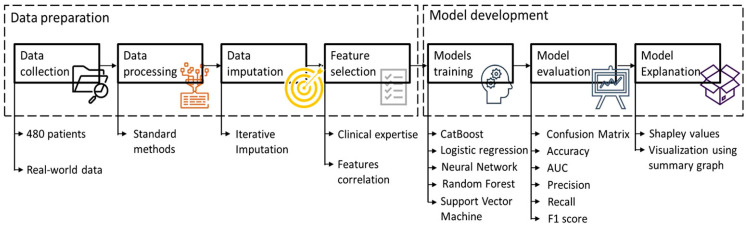
Representation workflow for developing different ML/explainable AI models. Reproduced from [159].

Beyond image analysis, multi-omics and clinical data integration using AI are crucial. Bayesian network models, described as ‘white-box’ models, offer accurate and interpretable predictions of immunotherapy responses in NSCLC, addressing the limitations of single predictive factors [151]. Explainable AI and ML methods have been developed using RWD from patients with advanced NSCLC to predict immunotherapy efficacy [159]. One such study involved 480 patients, with 407 receiving immunotherapy (Figure 13) [159]. An inflammatory response signature score model, using the least absolute shrinkage and selection operator computational algorithm, was established to predict responses to cancer immunotherapy and patient survival in a pan-cancer analysis that included NSCLC [160]. While primarily demonstrated in triple-negative breast cancer, the integration of whole-exome sequencing and scRNA-seq data with a spatial Quantitative System Pharmacology model to predict neoantigen burden and immunotherapy response exemplifies a sophisticated multi-omics modeling approach that could be extended to lung cancer [161].

Despite significant progress, challenges remain in the application of AI for predicting immunotherapy outcomes in lung cancer [150]. Future directions emphasize the harmonization of molecular cancer biology and AI to develop more effective treatment strategies in NSCLC. Continued advancements rely on exploiting high-dimensional oncological data through AI approaches to foster precision immuno-oncology. As AI continues to evolve, its capacity to synthesize complex data from various sources promises to refine our understanding of treatment response and pave the way for increasingly personalized and effective immunotherapy for lung cancer patients.

### 6.3. Radiotherapy Planning and Optimization with Artificial Intelligence

The integration of AI into radiotherapy planning and optimization for lung cancer has garnered significant attention in recent years, driven by the potential to enhance treatment precision, personalize therapy, and improve clinical outcomes. The current research demonstrated a multifaceted approach encompassing advanced segmentation techniques, treatment planning automation, adaptive strategies, and decision support systems, all leveraging AI’s capabilities.

One prominent area of focus is the development of AI-driven segmentation methods to accurately delineate organs-at-risk and tumor volumes, which are critical for effective treatment planning. Liu et al. [162] introduced a cross-layer attention fusion network (CLAF-CNN) that employs a novel architecture combining cross-layer spatial attention maps with TELD-Loss to improve the segmentation accuracy of organs-at-risk in lung and nasopharyngeal cancers. Such precise segmentation is fundamental for minimizing radiation exposure to healthy tissues and optimizing dose delivery. Similarly, Said et al. [163] proposed an AI-driven lung segmentation framework optimized via genetic algorithms, emphasizing the importance of accurate lung delineation in early lung cancer diagnosis and subsequent treatment planning.

**Figure 14:**
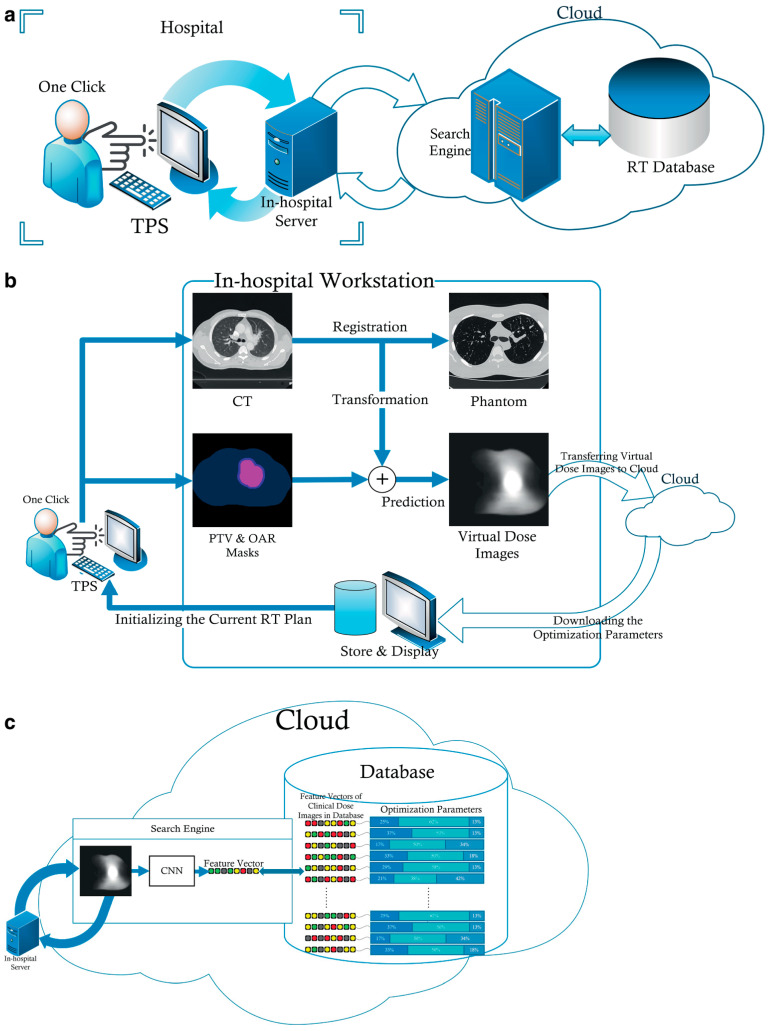
Architecture for dose-image agent retrieval: (**a**) αDiar’s internal workflow; (**b**) the processing workflow on the in-hospital server; (**c**) the corresponding workflow in the cloud. Reproduced from [165].

Beyond segmentation, AI has been instrumental in automating and enhancing treatment planning processes. Jiang et al. [164] investigated an AI-based automated planning system (MD Anderson Cancer Center AutoPlan system) for postmastectomy volumetric modulated arc therapy, demonstrating the potential for AI to generate high-quality plans efficiently. In the context of lung cancer, Shao et al. [165] proposed a knowledge-based planning system (αDiar) tailored for lung cancer patients treated with intensity-modulated radiotherapy (Figure 14), aiming to streamline plan generation while maintaining dosimetric quality. These systems exemplify how AI can reduce planning time and variability, leading to more consistent treatment quality.

Adaptive radiotherapy, which involves modifying treatment based on patient-specific changes over time, is another domain where AI shows promise. Tortora et al. [166] presented a deep reinforcement learning controller capable of optimizing daily dose fractions based on sequential CT scans during therapy for NSCLC. This approach enables personalized, dynamic treatment adjustments, potentially improving therapeutic efficacy and reducing toxicity. AI’s role extends to treatment planning optimization techniques that balance tumor coverage with organs-at-risk sparing. Duan et al. [167] explored a novel conformal radiotherapy and intensity-modulated radiation therapy combined planning technique for peripheral lung stereotactic body radiotherapy, which integrates the strengths of conformal and intensity-modulated approaches. The application of AI in beam angle optimization, as demonstrated by Fjellanger et al. [168], through the iCE system, further exemplifies efforts to systematically improve dose distribution by automating multi-criterial planning and beam selection, thereby enhancing plan quality and efficiency.

The incorporation of AI into clinical decision-making and prognosis prediction is also evident. Tang et al. [169] developed a radiomics-clinical AI model with a probability-weighted strategy to predict NSCLC prognosis, facilitating personalized treatment strategies. Similarly, Li [170] employed neural networks to predict lung dose-volume parameters based on tumor morphology and position, aiding in risk assessment and treatment customization. These models underscored AI’s potential to inform clinical decisions beyond mere plan generation, integrating complex patient data for tailored therapy.

Recent advancements have also focused on integrating AI with emerging technologies such as magnetic resonance-guided radiotherapy. Ma et al. [171] reviewed the progress in magnetic resonance-guided radiotherapy, highlighting how AI-driven workflow optimization and remote treatment models are addressing current challenges in lung cancer management. Furthermore, Gao et al. [172] introduced the Automated Iterative RTPlanning system, a scalable AI framework for generating high-quality treatment plans at scale, emphasizing the importance of large, standardized datasets for training robust AI models.

The human-AI interaction aspect, explored by Niraula et al. [173], emphasized the importance of transparency and collaborative decision-making in AI-assisted radiotherapy. Their study revealed that the effectiveness of AI tools depends on multiple factors, including prior knowledge, patient status, and model transparency, which are crucial considerations for clinical implementation. Finally, the broader landscape of AI in lung cancer radiotherapy encompasses diagnostic and therapeutic applications. Wang et al. [174] reviewed AI’s role across the entire spectrum, from screening to therapy, highlighting its strengths in improving accuracy and efficiency. The recent development of GPT-4Vision-guided treatment planning [175] exemplified frontier AI models’ potential to revolutionize clinical workflows by integrating reasoning capabilities with radiation oncology expertise.

In summary, the literature underscored the transformative potential of AI in lung cancer radiotherapy planning and optimization. From precise segmentation and automated plan generation to adaptive strategies and decision support, AI technologies are poised to enhance treatment personalization, improve clinical outcomes, and streamline workflows. However, challenges related to data quality, model transparency, and clinical integration remain, necessitating ongoing research and validation to fully realize AI’s benefits in this domain.

### 6.4. Artificial Intelligence Powered Clinical Trial Matching and Optimization

The integration of AI into lung cancer clinical trial matching and optimization has garnered significant attention in recent years, driven by the need to enhance patient selection, streamline trial processes, and improve therapeutic outcomes. The current research underscored the transformative potential of AI technologies in addressing longstanding challenges in oncology clinical trials, particularly in the context of lung cancer.

One of the foundational steps in leveraging AI for trial matching involves the formalization of clinical trial eligibility criteria. Mai et al. [176] developed a phenotyping pipeline that utilizes NLP to convert unstructured eligibility text into computable criteria within EHRs. This approach facilitated high-throughput cohort selection by enabling automated identification of eligible patients, thereby reducing manual effort and potential errors. Such formalization is crucial for scalable AI applications, as it ensures that complex eligibility parameters are accurately captured and interpreted by algorithms.

Building upon this, Lee et al. [177] proposed an automated pipeline for phenotyping eligibility criteria from EHRs, aiming to match patient characteristics with trial requirements. Their work emphasized the importance of integrating clinical data with AI-driven phenotyping to improve the precision of patient-trial matching. Similarly, Wang et al. [178] conducted a systematic review comparing standalone DL models with expert assessments for lung cancer diagnosis on chest CT scans. Their findings highlighted the growing role of AI in diagnostic accuracy, which indirectly supports trial matching by ensuring that patients are correctly diagnosed and staged, thus aligning them with appropriate trial eligibility.

The development of AI-powered platforms specifically designed for trial matching has been exemplified by Klein et al. [179], who described MatchMiner, an open-source platform that facilitates precision medicine trial matching. The platform employs AI algorithms to match genomic alterations and cancer types to trial eligibility criteria, offering both patient-centric and trial-centric modes. The authors report that MatchMiner has facilitated over 250 trial consents, demonstrating its practical utility in real-world settings. This platform exemplified how AI can operationalize complex eligibility matching, especially when combined with genomic data, which is particularly relevant in lung cancer where molecular profiling guides targeted therapies.

In the context of lung cancer, personalized treatment navigation and trial matching services like LungMATCH have been developed to address unmet needs. Saez et al. [180] described LungMATCH as a program that collects patient-reported biomarker testing, treatment history, and trial participation data to support personalized navigation. Although not solely AI-driven, such systems can be integrated with AI modules to enhance matching accuracy and efficiency, especially by incorporating real-time data and patient preferences.

AI’s role extends beyond mere matching to optimizing trial enrollment and management. Cesario et al. [181] introduced the Digital Research Assistant, a platform designed to support clinicians by providing real-time information on available trials. The Digital Research Assistant aimed to streamline patient enrollment processes, which is critical in lung cancer trials where timely enrollment impacts outcomes. By automating trial reporting and patient-trial matching, AI-powered tools like Digital Research Assistant can reduce delays and improve trial accrual rates.

Furthermore, AI’s application in pathology and biomarker assessment enhances trial selection precision. Baxi et al. [182] demonstrated the utility of AI in quantifying PD-L1 expression, a key biomarker for immunotherapy eligibility in lung cancer. Their large-scale retrospective analysis showed that digital pathology algorithms could identify patients who might benefit from immuno-oncology treatments more effectively than manual assessments. Similarly, Zenke et al. [183] validated an AI-powered PD-L1 analyzer, confirming its concordance with pathologists’ assessments. Accurate biomarker evaluation is essential for stratifying patients in trials, and AI-driven pathology tools can standardize and expedite this process.

**Figure 15:**
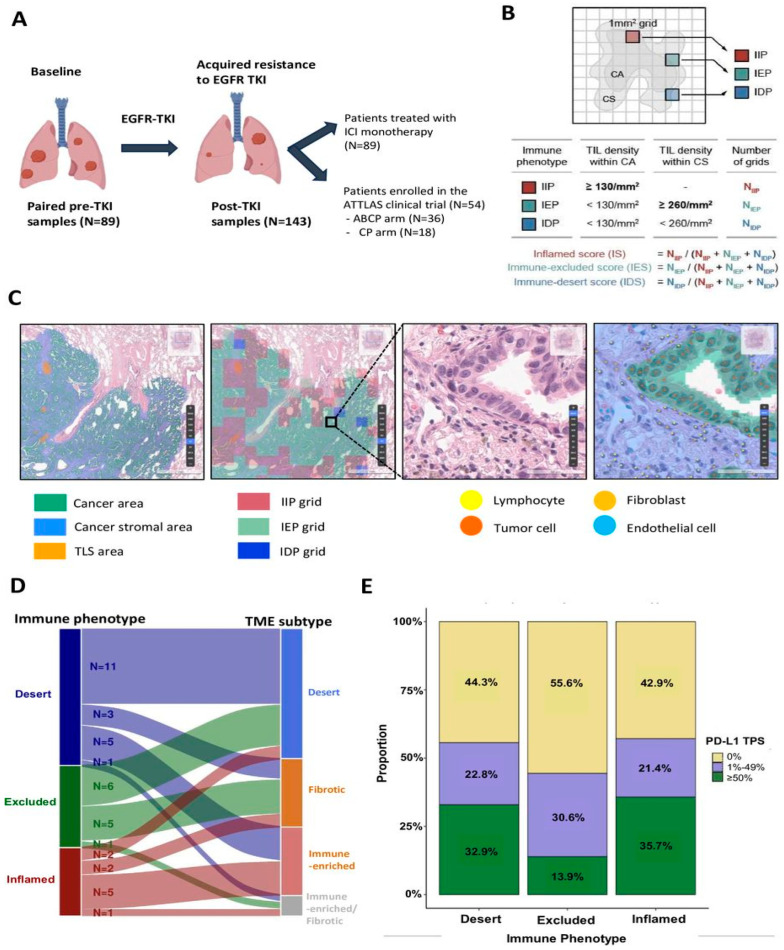
Design of the AI-powered tumor microenvironment analyzer and the study’s workflow. The components are as follows: (**A**) the study flow diagram, (**B**) the landscape of the AI-based immune phenotype analysis, (**C**) representative images from the tumor microenvironment analyzer, (**D**) an alluvial plot, and (**E**) a proportional bar plot showing PD-L1 expression scores by immune phenotype. Reproduced from [185].

AI also contributes to the evaluation of treatment responses and tumor microenvironment analysis. Dacic et al. [184] utilized ML models to assess pathologic responses after neoadjuvant therapy in NSCLC. Bang et al. [185] employed AI-powered spatial analysis to study tumor microenvironment changes in NSCLC patients with acquired resistance to EGFR-tyrosine kinase inhibitors (Figure 15). These insights can inform trial design by identifying biomarkers of resistance or response, thereby refining patient selection criteria. In addition to diagnostic and biomarker applications, AI supports clinical decision-making and trial management. Crimini et al. [186] compared rule-based, AI-powered, and manual trial matching tools, demonstrating that AI-based systems like OncoSolver can improve matching accuracy and efficiency. Such tools can be integrated into clinical workflows to assist clinicians in identifying suitable trials rapidly, which is particularly valuable in lung cancer where rapid disease progression necessitates swift action.

The impact of AI-driven trial participation on clinical outcomes has also been explored. Jung et al. [187] reported that lung cancer patients with EGFR-wild and ALK-negative tumors who participated in clinical trials experienced survival benefits compared to standard care. This underscored the importance of effective trial matching systems in increasing trial enrollment and, consequently, improving patient outcomes.

Finally, the overarching theme across these studies is the recognition that AI can significantly enhance the entire spectrum of lung cancer clinical trial processes—from eligibility criteria formalization and patient phenotyping to biomarker assessment, trial matching, and outcome prediction. The integration of AI tools like MatchMiner, Digital Research Assistant, and AI-powered pathology analyzers exemplifies a move toward more precise, efficient, and personalized trial management. As Le et al. [188] and Debellotte et al. [189] highlight, ongoing advancements in AI are poised to further accelerate drug discovery, early detection, and treatment optimization in lung cancer, ultimately leading to more effective and accessible clinical trials.

In summary, the current literature demonstrated that AI-powered clinical trial matching and optimization hold immense promise for lung cancer. These technologies facilitate more accurate patient selection, reduce trial enrollment barriers, and enable personalized treatment approaches. Continued innovation and integration of AI into clinical workflows are essential to realize the full potential of precision oncology in lung cancer, ultimately improving patient outcomes and advancing therapeutic development.

### 6.5. The Concept of Digital Twins and In-Silico Clinical Trials

**Figure 16:**
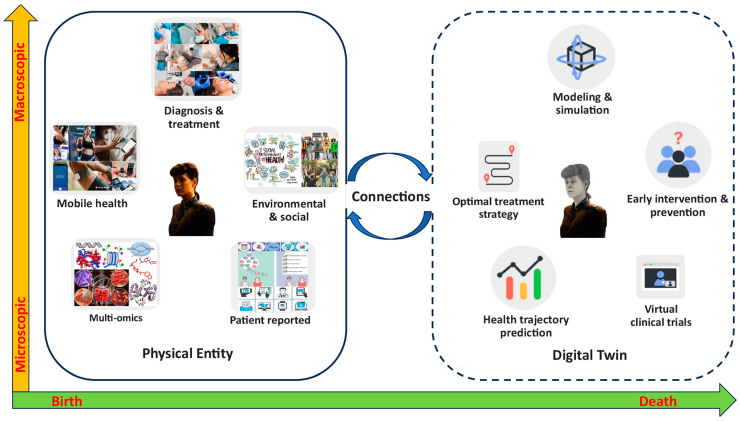
Overview of digital twin role. Reproduced from [190].

The beginning of AI-driven digital replicas of patients, commonly referred to as ‘digital twins’, has emerged as a transformative approach in the field of lung cancer treatment and clinical trial simulation (Figure 16). These virtual models serve as sophisticated representations of individual patients, enabling clinicians and researchers to simulate disease progression, predict treatment outcomes, and conduct virtual clinical trials with unprecedented precision and efficiency.

One of the primary motivations behind developing digital twins in healthcare is the significant underrepresentation of cancer patients in traditional clinical trials. According to a recent scoping review, less than 10% of adult cancer patients participate in such trials, which hampers the development of personalized therapies and delays the translation of research into clinical practice [189]. Digital twins offer a promising solution by facilitating virtual trials that can simulate the effects of various treatment regimens on individual patient models, thereby expanding access and reducing the logistical barriers associated with conventional trials.

In the context of lung cancer, advances in digital twin technology have demonstrated considerable potential. For instance, the Jinkō platform has been utilized to create virtual simulations aimed at predicting outcomes in lung cancer trials. These simulations enable researchers to test multiple treatment scenarios rapidly, assess potential responses, and optimize therapeutic strategies before applying them to real patients [191]. Such virtual trials not only accelerate the drug development process but also enhance the precision of treatment planning by accounting for patient-specific variables.

AI-powered models further enhance the fidelity of digital twins. A recent review highlighted the development of lung cancer prediction models that leverage biomedical large language models fine-tuned to forecast clinical outcomes from datasets involving NSCLC and intensive care unit patients [192]. These models utilize ML algorithms to analyze complex biomedical data, enabling the generation of highly accurate virtual replicas that reflect the unique biological and clinical characteristics of individual patients. This level of personalization is crucial for simulating treatment responses and predicting potential adverse effects.

The creation of highly accurate virtual replicas allows clinicians to conduct in silico experiments, testing various treatment options and observing potential outcomes without exposing patients to unnecessary risks. A comprehensive review emphasized that digital twins can simulate tumor growth, treatment effects, and disease progression, providing a dynamic platform for personalized medicine [193]. Such simulations are particularly valuable in lung cancer, where tumor heterogeneity and patient variability pose significant challenges to effective treatment.

Furthermore, digital twin technology is increasingly integrated with Internet of Things devices and biomedical data sources, enabling continuous monitoring and real-time updates of the virtual models. This integration facilitates adaptive treatment strategies that can respond to changes in patient condition, thereby improving clinical outcomes [194]. The ability to dynamically update the digital twin based on RWD enhances its utility in both clinical decision-making and virtual trial simulations.

In addition to predictive modeling, digital twins are being employed to simulate tumor behavior and treatment responses, aiding in the development of novel therapies. A systematic review of AI as a digital twin for prostate cancer care underscored the potential of these models to predict tumor growth and treatment efficacy, which can be extrapolated to lung cancer scenarios [195]. Such predictive capabilities are instrumental in designing more effective clinical trials and personalized treatment plans.

The future prospects of digital twins in lung cancer treatment are promising. They are envisioned to serve as comprehensive platforms that integrate genomic, imaging, and clinical data to facilitate precision medicine. By enabling virtual testing of treatment options, digital twins can reduce the time and cost associated with drug development and clinical trials, ultimately leading to more timely and tailored therapies for patients [196]. Moreover, the continuous evolution of AI algorithms and data integration techniques is expected to further enhance the accuracy and applicability of these virtual models.

In summary, AI-driven digital replicas of patients represent a significant advancement in lung cancer treatment and clinical trial methodology. They offer a means to simulate disease progression, predict treatment outcomes, and conduct virtual trials that are more inclusive, efficient, and personalized. As research progresses, these digital twins are poised to become integral tools in precision oncology, improving patient outcomes and accelerating the development of novel therapies.

## 7. From Algorithm to Clinic: Challenges in Translation and Validation

The field of AI holds immense promise for revolutionizing lung cancer diagnosis, prognosis, and treatment planning, addressing critical challenges such as early detection and precise characterization of the disease [197]. Despite significant interest and investment, translating these advanced algorithms from research environments into routine clinical practice remains a complex endeavor, fraught with numerous challenges [198]. This section presents current research on AI applications in lung cancer and critically examines the hurdles in their translation and validation, drawing exclusively from the recent literature.

**Figure 17:**
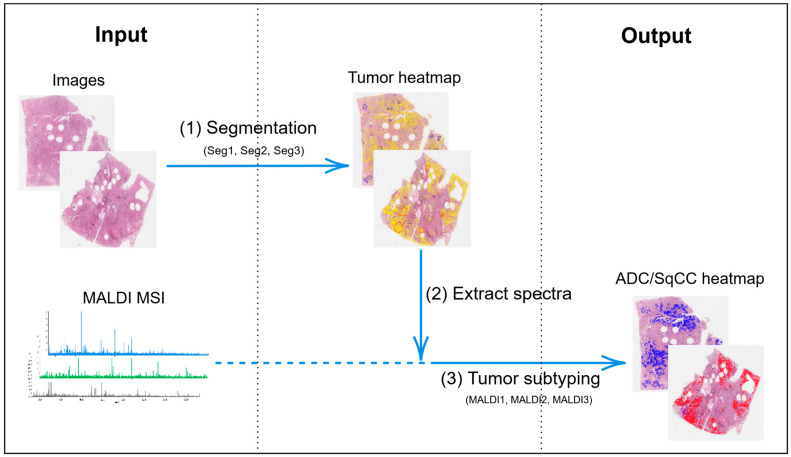
A summary of the classification algorithm, featuring the trained networks (Seg1 to 3 and MALDI1 to 3). Reproduced from [200].

AI applications in lung cancer span diverse modalities and clinical problems. In digital pathology, AI is being developed for tasks such as multi-class tissue segmentation in NSCLC, including adenocarcinoma and squamous cell carcinomas, providing a foundation for multiple downstream applications and patient care optimization [75,199]. Algorithms can detect and subtype tumor areas by combining information from digital microscopy WSIs and matrix-assisted laser desorption/ionization-mass spectrometry imaging of lung tissue sections (Figure 17) [200]. Automated systems can also quantify cell-level PD-L1 expression in NSCLC WSIs, a crucial biomarker, by detecting negative and positive tumor cells [201]. Furthermore, AI has been applied to predict lung cancer relapse from histopathology, incorporating uncertainty estimation to enhance clinical utility [116]. The development of large archival databases of digital pathology images is expected to further facilitate AI advancements in this area [202].

Beyond histopathology, AI leverages radiological characteristics from modalities like low-dose CT and chest CT scans. For instance, AI aids in the challenging task of identifying malignant from benign pulmonary nodules, a significant issue given the dramatic rise in indeterminate nodules due to low-dose CT screening [203,204]. Models based on DNA methylation biomarkers (PTGER4, RASSF1A, and SHOX2) and radiological characteristics have been developed to improve this distinction [203]. AI-based semantic segmentation algorithms applied to chest CT scans can even enable semi-automated lung cancer surgery planning by recognizing anatomical variants of pulmonary vessels [205]. Early warning models for lung cancer have also been constructed using ML on somatic mutation data, specifically by extracting top somatic mutation-related genes from the TCGA database [206]. The integration of liquid biopsy with AI classifiers is improving early lung cancer diagnosis, specifically for evaluating pulmonary nodules [204]. Looking ahead, multimodal DL frameworks combining CNN with DNN are proposed to enhance early-stage lung cancer prediction, aiming to overcome the challenge of subtle early radiological features [201]. Novel approaches extend to non-invasive diagnosis through exhaled breath analysis using surface-enhanced Raman spectroscopy, though distinct diagnosis between lung cancer and gastric cancer based on the same biomarkers (e.g., aldehydes) in breath remains a challenge [207]. Capsule networks with dynamic routing algorithms are also being optimized for classification of benign and malignancy in lung cancer using computed tomography images, noted for their robustness with relatively little training data [208].

Significant efforts have been directed towards validating these AI tools, often aiming for pathologist-level performance. For example, a large histopathology competition, PANDA, involving over a thousand developers, validated diverse AI algorithms for Gleason grading in prostate cancer, demonstrating pathologist-level performance on independent cross-continental cohorts [209]. Similarly, DL algorithms for PD-L1 expression detection in NSCLC were evaluated at both cell-level and slide-level, showing agreement with multiple pathologists on multi-centric, multi PD-L1 assay datasets [210]. DL-based AI for prostate cancer detection at biparametric magnetic resonance imaging was trained, validated, and tested using magnetic resonance imaging scans from 2 institutions [211]. Clinical validation studies have shown high sensitivity (1.0) and specificity (up to 0.969) for AI tools in tumor detection in colorectal biopsy specimens, even across multiple external cohorts [212]. Retrospective validation studies have also compared AI algorithm performance to that of surgeons in tasks like lung cancer surgery planning [205]. The exploration of uncertainty quantification within AI models is seen as an avenue to further improve performance and clinical application by providing clinicians with confidence alongside predictions [116].

Despite these promising developments, the clinical translation of AI in lung cancer faces considerable challenges. A primary barrier is the absence of widespread translation from research to deployed AI solutions [198]. One fundamental issue is the lack of standardized data acquisition protocols, which can limit algorithmic robustness and generalizability [213]. Furthermore, critical reporting standards are often overlooked; for example, ethnicity was frequently under-reported (81%) in a systematic review of AI in breast cancer, and model calibration was almost universally not reported (99%) [214]. This lack of transparency and completeness in reporting hinders reproducibility and confidence in AI models.

Algorithmic limitations present another set of difficulties. Developers face specific challenges when training neural networks on complex lung cancer tissue data, as identified in multimodal subtyping studies [200]. The subtle nature of early-stage radiological features makes accurate early detection a persistent challenge for AI models [201]. Distinguishing between different cancer types using shared biomarkers, such as aldehydes in exhaled breath for lung and gastric cancer, also poses a significant hurdle [207]. Moreover, AI models can exhibit limitations in robustness and generalizability, particularly when not trained and validated across diverse, multi-center cohorts [213].

Low levels of clinical acceptance represent a significant barrier to widespread adoption [213]. This can stem from a lack of confidence in AI predictions, especially without mechanisms like uncertainty quantification to convey model confidence to clinicians [116]. The potential for overdiagnosis, as seen with the increase in indeterminate pulmonary nodules following low-dose CT screening, underscores the need for highly accurate and reliable diagnostic tools [204]. Finally, barriers to effective interdisciplinary collaboration between AI developers, clinicians, and researchers also impede the translation process [213].

To address these multifaceted challenges, researchers proposed several strategies. A vertically integrated approach to AI development is advocated, incorporating early, cross-disciplinary consideration of impact evaluation, data lifecycles, and AI production [198]. This includes unifying norms for medical data collection, optimizing fractal algorithms, and deeply integrating AI with big data [213]. Multi-center clinical validation is crucial for demonstrating robustness and generalizability across diverse patient populations and clinical settings [213]. Developing and deploying user-friendly visual diagnostic software is essential to facilitate clinical adoption [213]. Moreover, cultivating interdisciplinary talents who can bridge the gap between theoretical mathematics, computer science, and clinical medicine is vital for successful translation [213]. Continued research into improving the performance and clinical application of models, potentially through techniques like uncertainty quantification, is also critical [116].

In summary, while AI in lung cancer diagnostics and prognostics shows tremendous potential through applications ranging from digital pathology to liquid biopsy and breath analysis, its journey from algorithm to clinic is marked by significant hurdles (Table 6). Challenges include the need for standardized data acquisition protocols, robust and generalizable algorithms, comprehensive validation across diverse cohorts, improved reporting standards, and overcoming barriers to clinical acceptance and interdisciplinary collaboration. Addressing these challenges through strategic, integrated approaches and fostering collaboration is paramount to realizing the full transformative impact of AI in lung cancer care.

## 8. Critical Appraisal of Reported AI Performance

The preceding sections highlight numerous studies where AI models demonstrate remarkably high diagnostic accuracy, area under the curve, or predictive performance for tasks such as nodule classification, mutation prediction, and immunotherapy response forecasting. While these results are promising and illustrate the technical potential of AI, a critical appraisal of the underlying evidence is essential to contextualize their clinical readiness. Key methodological limitations frequently temper the real-world applicability of these reported metrics.

### 8.1. Dataset Characteristics and Generalizability

Many of the cited high-performing models are developed and validated on retrospective, single-institution datasets [23,57,92]. These cohorts are often limited in size (frequently n < 1000) and may not capture the full spectrum of patient demographics, imaging equipment, acquisition protocols, or disease manifestations encountered in broader clinical practice. For instance, a model achieving 99% accuracy on a curated dataset from a specialized cancer center may perform significantly worse when applied to community hospital data or screening populations with a lower disease prevalence and a higher proportion of indeterminate findings. Performance can be artificially inflated in studies with a high-class imbalance or an enriched case mix that does not reflect the intended use population.

### 8.2. Validation Rigor and Overfitting

The distinction between internal validation (e.g., cross-validation on a single dataset) and robust external validation is crucial. A significant proportion of the literature reports only internal validation metrics, which are susceptible to overfitting and do not adequately assess model generalizability. While some challenges and multi-institutional studies have begun to address this [58,209], the field still lacks widespread adoption of prospective, externally validated trials. Furthermore, detailed reporting of calibration metrics—how well predicted probabilities align with observed outcomes—is rare [214], yet is vital for clinical decision-making where risk stratification is key.

### 8.3. Reporting Gaps and Reproducibility

Systematic reviews have identified frequent omissions in critical reporting elements for AI studies in oncology, including insufficient description of patient eligibility, handling of missing data, and details of model training and tuning [214,215]. The under-reporting of participant demographics, particularly ethnicity, raises concerns about algorithmic bias and the equitable performance of tools across diverse populations. Without complete and transparent reporting according to guidelines such as TRIPOD-AI or STARD-AI, the reproducibility and independent verification of results remain challenging.

In summary, the exceptional performance metrics reported in many studies represent a compelling proof of concept. However, their translation into reliable clinical tools depends on moving beyond optimized performance on constrained datasets. The path forward requires a concerted emphasis on large, diverse, multi-institutional datasets, rigorous external and prospective validation, adherence to transparent reporting standards, and ultimately, demonstration of improved clinical outcomes in randomized controlled settings. The subsequent sections delve deeper into these overarching challenges of clinical integration, interpretability, and ethical deployment.

## 9. Interpretability and Explainability of Artificial Intelligence Models

The interpretability and explainability of AI models in healthcare, particularly in lung cancer diagnosis, have garnered significant attention in the recent literature. As AI systems become increasingly integrated into clinical workflows, understanding the rationale behind their recommendations is crucial for fostering trust, ensuring safety, and facilitating clinical decision-making. Several methods, notably SHapley Additive exPlanations (SHAP) and Local Interpretable Model-agnostic Explanations (LIME), have emerged as prominent tools to elucidate AI model outputs, especially in complex domains like lung cancer detection.

**Figure 18:**
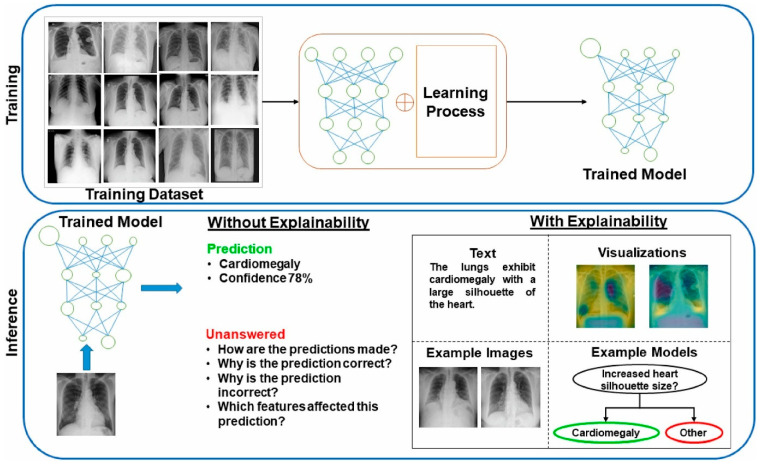
Explainable AI helps understand the model’s decision. Reproduced from [218].

A comprehensive review of explainable AI techniques in healthcare underscored the importance of these methods in making AI models more transparent (Figure 18) [216]. Both SHAP and LIME are frequently cited as the most commonly used local explanation techniques, capable of providing insights into individual predictions [217]. These methods serve to bridge the gap between the often ‘black box’ nature of DL models and the need for clinicians to understand the underlying decision processes. For instance, in lung cancer detection, DeepXplainer—a DL based approach—utilizes explainability techniques to enhance interpretability, thereby aiding clinicians in understanding model recommendations [216].

SHAP, rooted in cooperative game theory, assigns each feature an importance value that reflects its contribution to the model’s output [217]. Its ability to provide consistent and locally accurate explanations makes it particularly suitable for high-stakes medical applications. Similarly, LIME offers local fidelity by approximating complex models with interpretable surrogate models around specific instances [217]. These methods are model-agnostic, meaning they can be applied across various AI architectures, including deep neural networks, which are prevalent in lung cancer diagnosis [219].

The literature emphasized that these explanation techniques not only improve transparency but also enhance clinicians’ trust in AI systems. By providing clear, feature-level insights, SHAP and LIME help clinicians understand why a particular diagnosis or recommendation was made, which is vital for clinical acceptance [219]. For example, in lung cancer detection, explanations generated by SHAP can highlight the most influential features—such as nodule size, shape, or texture—that contributed to the model’s decision, aligning with clinical reasoning processes [216].

Furthermore, the integration of explainability methods like SHAP and LIME into AI models supports the development of more interpretable and generalizable systems. An illustrative example is the use of these techniques alongside AutoML and counterfactual analysis to improve model transparency and robustness in disease prediction tasks, including lung cancer [220]. Such approaches demonstrate that combining explainability with advanced ML techniques can lead to models that are both accurate and interpretable, facilitating their adoption in clinical settings.

Despite their advantages, the application of SHAP and LIME in healthcare faces certain challenges. The complexity of explanations, computational costs, and the potential for misinterpretation are noted as limitations [221]. Nonetheless, ongoing research aims to refine these methods to better suit clinical needs. For instance, visual explanations—such as heatmaps or feature importance plots—are employed to make the explanations more intuitive for clinicians [222]. These visual cues help clinicians quickly grasp the rationale behind model predictions, which is particularly useful in high-pressure environments like lung cancer diagnosis.

The role of explainability extends beyond individual predictions to broader clinical trust and system adoption. As highlighted in recent reviews, explainable AI methods like SHAP and LIME are instrumental in fostering clinician confidence, ensuring that AI recommendations are transparent and justifiable [223]. This is especially critical in lung cancer diagnosis, where early detection significantly impacts patient outcomes. The ability of these methods to provide localized explanations aligns well with the need for personalized medicine, allowing clinicians to understand the specific factors influencing each case.

In summary, the literature consistently underscored the importance of explainability techniques such as SHAP and LIME in making AI models more interpretable in the context of lung cancer diagnosis. These methods facilitate understanding of model decisions at a granular level, thereby enhancing clinician trust and supporting clinical decision-making (Table 7). While challenges remain, ongoing advancements in explainability are poised to improve the integration of AI into healthcare, ultimately leading to more transparent, trustworthy, and effective diagnostic tools.

## 10. Ethical, Legal, and Social Implications

The integration of AI into lung cancer diagnosis and treatment presents a complex landscape of ethical, legal, and social implications (ELSI). As AI technologies become increasingly prevalent in healthcare, particularly in oncology, understanding these multifaceted challenges is crucial for ensuring responsible development and deployment. The literature underscored that while AI offers significant potential to enhance diagnostic accuracy, treatment efficacy, and operational efficiency, it simultaneously raises critical concerns that must be addressed through comprehensive ethical and legal frameworks.

One of the primary ethical considerations in AI applications for lung cancer is the issue of bias and fairness. Oyeniran et al. [224] emphasized that bias in ML models can lead to unfair treatment outcomes, which is particularly problematic in sensitive areas like cancer diagnosis where disparities can have life-altering consequences. Addressing bias is essential not only for ensuring equitable healthcare but also for maintaining trust among diverse patient populations. Legal implications are equally significant, especially regarding data ownership, privacy, and liability. The review by Chamouni et al. [225] explicitly discussed the legal challenges associated with AI in lung cancer, including issues of data confidentiality, informed consent, and accountability for diagnostic errors. The legal landscape must evolve to clarify responsibilities among developers, healthcare providers, and regulators, ensuring that legal standards keep pace with technological advancements. The importance of establishing clear legal guidelines is echoed by Roche et al. [226], who advocated for policies that incorporate diverse social and cultural perspectives to develop ethically sound AI standards.

Trustworthiness and governance of AI systems are central themes in the literature. Zhang and Zhang [227] proposed a multidisciplinary approach to ensure the trustworthiness of medical AI, emphasizing data quality, algorithmic bias, opacity, safety, security, and responsibility attribution. They advocated for ethical governance frameworks that incorporate principles such as transparency, accountability, and fairness, which are vital for fostering patient and clinician confidence in AI-driven diagnostics and treatments. Mökander et al. [228] further contributed by outlining auditing mechanisms as governance tools to evaluate AI systems systematically, ensuring they adhere to ethical and legal standards.

Social implications, including issues of equity, access, and societal trust, are also prominent. The work by Sun [229] explored how AI can optimize social welfare systems, including healthcare, but notes that ethical challenges such as algorithmic bias and data privacy must be carefully managed to prevent exacerbating existing disparities. Similarly, Mayrhofer et al. [230] underscored the importance of balancing innovation with societal values, emphasizing trust-building and gender-sensitive approaches in biomedical research.

The ethical principles of autonomy, beneficence, nonmaleficence, and justice serve as foundational guides in addressing these challenges. Gagne et al. [231] adopted a multidisciplinary perspective to propose ethical governance measures that uphold these principles in medical AI, including in lung cancer applications. They argued that ensuring data quality, minimizing bias, and clarifying responsibility are essential for aligning AI practices with ethical norms. Cyberethics, further emphasizes the importance of respecting patient autonomy and confidentiality in the digital age [231].

Practical implementation of ethical AI requires translating theoretical principles into actionable strategies. Bleher et al. [232] explored how different AI ethics approaches conceptualize this translation, highlighting the need for continuous improvement and proactive measures to embed ethics into AI development processes. Auditing, as a governance mechanism, is particularly relevant; Mökander et al. [228] proposed a 3-layered auditing approach to evaluate large language models, which can be adapted for AI systems used in lung cancer diagnostics.

In summary, the literature collectively underscored that the deployment of AI in lung cancer care must be accompanied by robust ethical, legal, and social safeguards (Table 8). Addressing bias, ensuring transparency, clarifying liability, and respecting patient rights are fundamental to fostering trust and ensuring equitable access. Developing comprehensive governance frameworks, informed by multidisciplinary insights and continuous auditing, is essential for aligning AI innovations with societal values and legal standards. As AI continues to evolve, ongoing dialogue among stakeholders—including clinicians, policymakers, patients, and ethicists—is vital to navigate the complex ELSI landscape and realize the full potential of AI in lung cancer treatment responsibly.

## 11. Economic Impact and Cost-Effectiveness of Artificial Intelligence Integration

The integration of AI into lung cancer diagnosis, biomarker discovery, and drug development has emerged as a transformative force with significant economic implications. The current literature underscored the potential of AI to enhance cost-effectiveness and improve clinical outcomes, thereby influencing healthcare economics profoundly.

One of the primary economic benefits of AI integration in lung cancer management is its capacity to facilitate rapid and accurate diagnosis through multimodal data analysis. According to recent studies, multimodal AI systems are capable of integrating heterogeneous datasets, including imaging, clinical, and molecular data, into cohesive analytical frameworks [233]. This integration not only improves diagnostic precision but also reduces the time and resources required for traditional diagnostic procedures. For instance, AI-driven non-invasive diagnostic methods that combine imaging and clinical markers have been shown to streamline the diagnostic process, potentially lowering costs associated with invasive procedures and misdiagnoses [234].

Grenier et al. [235] discussed the potential role of AI algorithms in lung cancer screening using low-dose CT. They emphasize that AI can serve as a second reader, potentially reducing false positive rates, which are a significant concern in screening initiatives. This reduction could translate into cost savings by decreasing unnecessary follow-up procedures and biopsies, thereby improving the overall cost-effectiveness of screening programs. Similarly, Harpaz et al. [236] evaluate the cost-effectiveness of lung cancer screening in Australia, considering outcomes from trials like NLST and NELSON. Although their analysis does not explicitly focus on AI, the integration of AI-driven image interpretation could further refine screening accuracy and resource utilization, suggesting a pathway for AI to enhance economic efficiency in national screening strategies.

The application of AI extends beyond screening to diagnostic and prognostic domains. Wale et al. [237] reviewed the effectiveness of AI models, including ML and DL, in cancer diagnosis, indicating that AI models generally demonstrate favorable performance. These models can potentially streamline diagnostic workflows, reduce diagnostic delays, and improve early detection, which are critical for cost-effective management. Moreover, Safarian et al. [85] explored the role of AI-enhanced radiomics in PET/CT imaging, demonstrating that AI can improve tumor characterization and molecular marker detection. Such advancements can lead to more precise treatment selection, potentially reducing the costs associated with ineffective therapies and adverse events.

In the realm of treatment optimization, Bonci et al. [238] systematically reviewed the impact of AI, artificial neural networks, and ML models on patient outcomes and economic metrics. They highlighted that while AI has shown promise in improving prognosis and treatment decision-making, its impact on patient-reported outcomes and overall survival remains underexplored. Nonetheless, the potential for AI to personalize therapy and avoid unnecessary treatments could lead to significant cost savings, especially in complex cases like NSCLC. Additionally, the study by Zhu and Tan [239] on Chinese patent medicines combined with chemotherapy underscores the importance of evaluating cost-effectiveness in diverse treatment modalities, which could be further enhanced by AI-driven decision support systems.

The economic evaluation of AI-based interventions also faces methodological challenges. Fagery et al. [240] conducted a systematic review of health economic evidence for liquid biopsy assays, noting that few studies incorporate comprehensive economic modeling. They emphasized that the complexity of precision medicine, including AI applications, necessitates sophisticated models that can capture long-term costs and benefits. Similarly, Farah et al. [241] analyzed the suitability of health technology assessment frameworks for AI-based medical devices, suggesting that current models may need adaptation to adequately evaluate AI’s unique features, such as continuous learning and algorithm transparency.

Furthermore, AI’s role in biomarker discovery is pivotal in advancing personalized medicine for lung cancer. AI-based biomarkers derived from routine clinical data can enhance the accessibility of tailored treatment strategies, leading to more effective and targeted therapies [242]. The discovery and validation of tumor biomarkers are crucial for prognosis and treatment decisions, and AI accelerates this process by analyzing vast datasets to identify novel biomarkers with high predictive value [243]. The integration of multiple tumor biomarkers supported by AI enhances the precision of diagnosis and prognosis, which can translate into more efficient resource allocation and reduced expenditure on ineffective treatments [243].

In addition to diagnostics and biomarker discovery, AI significantly impacts drug development processes. The demand for faster, cost-effective drug discovery pipelines is increasingly being met through AI-driven approaches. AI facilitates the identification of promising drug candidates, predicts drug-target interactions, and generates novel chemical entities, thereby reducing the time and costs associated with traditional drug development [244]. This is particularly relevant in oncology, where the complexity of tumor biology necessitates sophisticated analytical tools. AI’s ability to analyze large-scale biological data accelerates the identification of therapeutic targets and the development of personalized treatment regimens, ultimately leading to more efficient use of healthcare resources [245].

Furthermore, proactive identification of high-risk populations through AI is emerging as a promising strategy. Ricketts et al. [246] described an AI-based approach to early lung cancer detection using retrospective population data, illustrating how AI can facilitate targeted screening and early intervention. Such strategies could improve cost-effectiveness by focusing resources on individuals most likely to benefit, thereby reducing unnecessary screening and associated costs.

Operational challenges and implementation considerations are also critical. Huang et al. [247] assessed the feasibility and cost-effectiveness of deploying mobile low-dose CT units integrated with AI diagnostics in underserved populations. Their findings suggested that AI can enhance accessibility and efficiency, especially in rural or resource-limited settings, potentially reducing disparities and associated costs. However, they also acknowledged operational hurdles, including infrastructure and training requirements, which must be addressed to realize AI’s economic benefits.

The economic impact of AI in lung cancer extends beyond individual patient care to hospital operations and healthcare systems at large. AI applications in hospital settings improve operational efficiency by optimizing workflows, resource allocation, and patient management [245]. These improvements can lead to cost savings by reducing hospital stays, minimizing unnecessary procedures, and streamlining clinical decision-making processes. Moreover, AI’s capacity to support clinical decision-making enhances treatment accuracy, potentially reducing the costs associated with adverse events and ineffective therapies [245].

The broader implications of AI’s economic impact are also discussed in the context of sustainable cancer care. AI-driven solutions contribute to more sustainable healthcare by supporting the development of cost-effective diagnostic and therapeutic strategies. For example, AI’s role in supporting biospecimen research and integrating various data types fosters the discovery of more effective biomarkers and targeted therapies, which can lead to better resource utilization and reduced long-term costs [248]. Finally, the concept of multimodal AI and its potential to reshape oncology practices is gaining prominence. Multimodal AI systems that integrate diverse data sources are poised to revolutionize lung cancer management by enabling more precise, personalized, and cost-effective care pathways [233]. This holistic approach not only improves clinical outcomes but also aligns with economic goals by reducing unnecessary interventions and optimizing treatment strategies.

In summary, the current literature highlighted that AI integration in lung cancer diagnosis, biomarker discovery, and drug development offers substantial economic benefits (Table 9). These include reduced diagnostic and treatment costs, accelerated drug development pipelines, and improved operational efficiencies within healthcare systems. As AI technologies continue to evolve, their capacity to deliver cost-effective, personalized, and sustainable lung cancer care is expected to expand, ultimately transforming the economic landscape of oncology healthcare.

## 12. Global Initiatives, Collaborations, and Benchmarking Challenges

The integration of AI into healthcare systems globally is increasingly seen as a crucial strategy for addressing persistent challenges such as escalating costs, limited access, and the growing demand for personalized care [33]. This transformative potential extends significantly to oncology, particularly in the complex domain of lung cancer, where global initiatives, international collaborations, and robust benchmarking methodologies are paramount. The commitment to harnessing AI is evident in global governmental assessments of AI readiness [249] and broad institutional collaborations across diverse sectors [250].

Significant global initiatives are underway to advance cancer research and treatment using AI. The European Cancer Moonshot Lund Center, for instance, has developed a comprehensive biobanking framework that merges rigorous sample handling, advanced automation, and multi-omics analyses. This strategy aimed to accelerate precision oncology, providing a foundational infrastructure for AI-driven research [251]. Similarly, the Biden Cancer Moonshot emphasizes the goal of ending cancer through intensified international collaboration and has even proposed a Global Cancer Fund, drawing inspiration from successful global health initiatives [252]. These large-scale endeavors underscore a collective commitment to leveraging global partnerships for scientific advancement.

Collaborative efforts are also reshaping how AI is developed and deployed in oncology. The Federated Tumor Segmentation initiative, involving 30 institutions globally, utilizes Federated Learning to address computational challenges and facilitate large-scale data analysis while maintaining data privacy [253]. This model represents a vital form of collaboration, allowing diverse institutions to contribute to and benefit from shared AI development. Beyond specific projects, international collaborations and partnerships, particularly between developed and developing countries, are considered essential for surmounting challenges in applying AI to global health issues, including lung and breast cancers [254]. Organizations like Stand Up To Cancer^®^ further exemplify this collaborative spirit through scientific initiatives that apply computational and AI techniques to model cancer growth, understand drug resistance, and discover new drug combinations to overcome treatment challenges [https://standuptocancer.org/what-we-do/scientific-initiatives/, accessed on 1 December 2025].

Within the realm of lung cancer specifically, AI-based digital pathology has been a prominent research trend over the past 2 decades. Investigations into this field have provided a comprehensive knowledge framework, highlighting research hotspots and gaps, particularly concerning its application in immunotherapy for lung cancer patients [254]. This focus on digital pathology underscores the intricate ways AI is being integrated into diagnostic and therapeutic pathways for lung cancer.

However, the effective deployment of AI solutions necessitates robust and fair benchmarking to ensure their reliability and efficacy. One direct example is the LUNA25 Challenge, which specifically aims to benchmark AI against radiologists for lung cancer screening in CT scans [255]. Such challenges are critical for validating AI’s performance in real-world clinical scenarios. The Federated Tumor Segmentation challenge, while focused on brain tumor segmentation, serves as another international competition designed to benchmark algorithms, involving a consortium of data contributors, participants, and organizers [256]. This approach highlights the complexity of developing fair decentralized benchmarking for healthcare AI, especially given the need for common datasets and evaluation standards across diverse institutions [256,257]. Establishing collaborative open-source platforms is crucial for facilitating this process, allowing AI to solve real-world problems using standardized evaluation criteria [257].

In summary, the landscape of AI in lung cancer is defined by a dynamic interplay of global initiatives, extensive collaborations, and dedicated efforts to overcome complex benchmarking challenges. From comprehensive biobanking strategies at the European Cancer Moonshot Lund Center and the ambitious goals of the Biden Cancer Moonshot to the federated learning frameworks of the Federated Tumor Segmentation initiative, the drive to harness AI’s potential is clear. International partnerships remain indispensable for advancing AI in global health, coupled with the continuous refinement of evaluation standards through benchmarking challenges like LUNA25 and collaborative platforms. These concerted efforts, building upon foundational research in areas like digital pathology for lung cancer, are crucial for transforming healthcare and addressing humanity’s greatest challenges in cancer care.

## 13. Limitations and Future Prospects

AI has shown significant promise in the management of lung cancer, offering advancements in early detection, diagnosis, and personalized treatment. However, several limitations hinder its full integration into clinical practice. These challenges include issues related to model generalizability, interpretability, and ethical considerations. Despite these hurdles, the future prospects of AI in lung cancer remain promising, with ongoing research aimed at overcoming current limitations and enhancing AI’s role in clinical settings.

- External validation and generalizability of AI models: Many AI models for lung cancer diagnosis and biomarker discovery lack extensive external validation across diverse populations and clinical settings. Conduct large-scale, multicenter prospective studies to validate AI models on heterogeneous datasets, including different ethnicities and imaging protocols. Develop standardized benchmarking datasets for reproducibility. Limited external validation restricts clinical adoption due to concerns about model robustness and applicability beyond initial training cohorts [152,215,258].
- Interpretability and explainability of multi-omics AI models: Multi-omics integration models often function as ‘black boxes,’ limiting clinical trust and interpretability. Develop interpretable AI frameworks that incorporate biological pathway knowledge and provide transparent decision-making processes, such as attention mechanisms and explainable AI tools. Enhancing interpretability bridges the gap between computational predictions and clinical decision-making, fostering trust and adoption [259,260,261].
- Data heterogeneity and integration challenges in multi-modal AI: Integration of heterogeneous data types (imaging, genomics, pathology, clinical) remains complex, with issues in data quality, missing values, and standardization. Design robust data harmonization pipelines and imputation methods; establish standardized protocols for multi-modal data collection and preprocessing; develop AI models resilient to missing or noisy data. Data heterogeneity and integration complexity hinder effective multi-modal AI model training and limit reproducibility [101,261].
- Limited sample sizes for multi-omics and multi-modal datasets: Small sample sizes in multi-omics and integrated datasets reduce statistical power and model robustness. Promote large-scale data sharing initiatives and consortia to aggregate multi-omics and imaging data; employ data augmentation and transfer learning techniques to mitigate sample size limitations. Small datasets increase overfitting risk and reduce generalizability of AI models, especially in complex multi-omics contexts [79,262,263].
- Ethical, privacy, and regulatory frameworks for AI in lung cancer: Ethical concerns, data privacy, and lack of clear regulatory guidelines impede clinical implementation of AI tools. Develop comprehensive ethical guidelines and privacy-preserving AI methods; engage regulatory bodies to establish standards for AI validation, transparency, and accountability in lung cancer care. Addressing ethical and regulatory challenges is essential for safe, equitable, and trustworthy AI deployment in clinical practice [152,263,264].
- Prospective clinical trials for AI-guided treatment personalization: Most AI applications in treatment personalization lack prospective clinical trial validation demonstrating improved patient outcomes. Design and conduct randomized controlled trials evaluating AI-guided treatment decisions, especially in immunotherapy and targeted therapy contexts, to assess clinical benefit and cost-effectiveness. Prospective evidence is critical to confirm AI’s impact on treatment efficacy and to support integration into clinical workflows [265,266].
- Standardization of biomarker discovery and validation: Variability in biomarker identification methods and lack of consensus on clinical utility limit translation of AI-discovered biomarkers. Establish standardized pipelines for biomarker discovery, validation, and reporting; integrate AI-derived biomarkers with clinical decision support systems for real-world testing. Standardization improves reproducibility and facilitates clinical adoption of AI-identified biomarkers [101,267,268].
- Addressing dataset bias and population diversity: AI models often trained on biased or homogeneous datasets, limiting performance across diverse patient populations. Curate diverse, representative datasets; implement bias detection and mitigation strategies in AI model development; evaluate model fairness across demographic subgroups. Mitigating bias is necessary to ensure equitable AI performance and avoid exacerbating health disparities [215,258,269].
- Computational and resource limitations of advanced AI techniques: Emerging AI methods like quantum ML face computational constraints and early-stage development challenges. Invest in scalable quantum computing infrastructure and hybrid quantum-classical algorithms; benchmark quantum AI against classical methods in lung cancer applications. Overcoming computational barriers is required to realize the potential advantages of novel AI paradigms [270].
- Integration of AI into clinical workflows and decision support: Lack of seamless integration of AI tools into existing clinical workflows limits usability and clinician acceptance. Develop user-friendly AI interfaces and decision support systems; conduct usability studies; train clinicians on AI interpretation and application; ensure interoperability with electronic health records. Effective integration enhances clinical utility and adoption of AI technologies in lung cancer care [258,271].

In summary, while AI in lung cancer presents numerous opportunities, it is crucial to address the existing limitations to fully realize its potential. The development of standardized methodologies, robust validation processes, and ethical frameworks will be essential in advancing AI’s role in lung cancer management. As research progresses, AI is poised to transform lung cancer care, offering improved diagnostic accuracy, personalized treatment options, and ultimately, better patient outcomes.

## 14. Conclusive Remarks

The literature indicated that AI has made substantial strides in transforming lung cancer care, particularly in the domains of diagnosis, biomarker discovery, and drug development. Advanced AI methodologies, including DL architectures such as CNNs and graph convolutional networks, have demonstrated high diagnostic accuracy in early detection of lung cancer, frequently matching or surpassing expert human performance. Radiomics and imaging-based AI models have been extensively validated to improve lung nodule classification and mutation prediction, thereby enhancing non-invasive molecular profiling. However, despite these promising results, challenges in model generalizability and external validation remain pervasive, limiting their immediate clinical translation.

AI’s integration with multi-omics data—encompassing genomics, transcriptomics, proteomics, and metabolomics—has enhanced biomarker discovery and molecular characterization of lung cancer subtypes. This multi-modal fusion facilitates a more comprehensive understanding of tumor heterogeneity and immune microenvironment interactions, which are critical for predicting therapeutic response, especially to immunotherapy. Frameworks that incorporate interpretability and explainability, such as attention-based models, have begun to bridge the gap between complex computational predictions and clinical decision-making, fostering greater clinician trust. Yet, issues with data heterogeneity, limited sample sizes for integrated omics datasets, and lack of standardized methodologies underscore the need for further refinement and harmonization.

In the field of treatment personalization and drug development, AI models have shown potential in optimizing therapy selection, prognosticating outcomes, and identifying novel therapeutic targets. Retrospective analyses demonstrate AI’s ability to uncover predictive biomarkers that can stratify patients for immunotherapy and targeted treatments, advancing precision oncology. Nevertheless, prospective clinical validation and incorporation into real-world treatment protocols are still scarce, and the dynamic evolution of tumor biology presents continuous challenges for AI model adaptability.

Finally, the literature recognized that clinical implementation of AI in lung cancer is tempered by ethical, practical, and regulatory challenges. Model interpretability, data privacy, algorithmic bias, and the need for transparent, explainable AI systems are paramount concerns. Multicenter collaborations, standardized evaluation frameworks, and regulatory guidelines are essential to overcoming these barriers. Taken together, the research underscores AI’s transformative potential in lung cancer care but highlights the necessity for rigorous validation, methodological standardization, and ethical integration to fully realize its benefits in precision medicine.

## Figures and Tables

**Table 1:** Key epidemiological statistics of lung cancer: global burden and trends.

Metric	Period	Value	Key Context
Global prevalence	2021	~3.25 million cases	Age-standardized rate: 37.3 per 100,000
Global incidence	2022	2.48 million new cases	Highest rates in Europe and Asia; ~8.5× higher in high-HDI vs. low-HDI countries
Global mortality	2022	~1.82 million deaths	Leading cause of global cancer mortality; age-standardized rate: 16.8 per 100,000
Mortality-to-incidence ratio	Global aggregate	0.71	Proxy for 5-year survival; indicates high fatality relative to diagnosis
Gender disparity	2020 data	Incidence and mortality ~2× higher in men vs. women	Pattern observed globally, though rates among women are rising in some high-income regions
Regional burden (mortality)	2020	Eastern Asia accounts for ~50% of global deaths	Hungary: highest mortality rate (42.4/100,000); Nigeria: lowest (0.86/100,000)
Historical trend (mortality increase)	1990–2019	+91.75%	Reaching >2 million deaths in 2019
Projected annual burden (2050)	2050 (projected)	3.8 million new cases; 3.2 million deaths	Driven by aging populations and persistent risk factors (e.g., smoking, air pollution)
Projected United States burden (2050)	2050 (projected)	~330,000 new cases; ~200,000 deaths	Notable gender disparity in incidence and mortality expected to persist

**Table 2:** Lung cancer challenges and the promise of AI.

Category	Key Challenge	Promise of AI	Relevant AI Applications	Refs.
Early detection and diagnosis	Lack of discernible symptoms in early stages; difficulty in early detection.	AI enhances screening efficiency and accuracy, enabling earlier diagnosis.	Nodule classification: AI improves classification of pulmonary nodules on CT scans. Radiomics and DL: Central to detection and diagnosis in lung imaging. Performance: AI can perform equivalently to an average radiologist in identifying tumors on chest radiographs.	[16,20,21]
Precise diagnosis and subtyping	Differentiating between subtypes (e.g., adenocarcinoma in situ vs. invasive) is complicated by tissue heterogeneity.	AI enables precise cancer subtyping and grading through computational pathology and advanced imaging analysis.	Computational pathology: DL models analyze whole-slide images for subtyping and grading. Multi-modal AI: Integrates radiological, clinical, and genetic data for more personalized diagnostic tools.	[22]
Biomarker and treatment response	Biomarkers like carcinoembryonic antigen are not specific to lung cancer; treatment resistance (e.g., to immunotherapy in NSCLC) is a major obstacle.	AI discovers non-invasive biomarkers and predicts treatment response, personalizing therapy selection.	Predicting EGFR status: AI algorithms using radiomics predict mutation status for targeted therapy. Immunotherapy response: AI-driven gene signatures (e.g., stemness-related) decipher prognosis and immunotherapy response. Precision immuno-oncology: AI exploits high-dimension data for predictive biomarker discovery.	[24,25,26]
Health equity and access	Systemic disparities in access to preventive services for ethnically and socioeconomically marginalized groups.	AI has the potential to improve research methods and bolster outcomes, addressing disparities in access.	Equity potential: Integration of robust AI models with diverse datasets holds promise for achieving equity across the diagnostic continuum.	[15]
Trust and adoption	‘Black box’ problem; lack of transparency in AI tools hinders critical medical judgment.	Development of explainable AI provides clarity and trustworthiness in predictions, fostering clinical adoption.	Explainable AI: A growing emphasis on explainable AI to ensure understandable insights into the AI decision-making process.	[19,23]

**Table 3:** Overview of core AI methodologies in medicine.

AI Methodology	Description	Key Applications in Medicine	Examples in Lung Cancer Context	Refs.
ML	Algorithms that learn patterns from data to make predictions or decisions without being explicitly programmed.	Clinical decision support, risk stratification, optimizing patient selection for clinical trials.	Predicting EGFR mutation status; classifying pulmonary nodules as benign or malignant.	[28,29]
DL	A subset of ML using multi-layered (deep) neural networks to model complex, hierarchical patterns from raw data.	Medical image analysis, automated segmentation, and feature extraction from radiology and pathology images.	Automated detection and characterization of lung nodules on CT scans; cancer subtyping from whole-slide images.	[30,31]
CNNs	A specialized DL architecture designed for processing grid-like data such as images, using convolutional layers to detect spatial features.	Radiology and pathology image classification, detection, and segmentation.	Identifying malignant nodules on CT; Gleason grading in pathology; reducing radiologist reporting burden.	[31,32]
NLP	Techniques for analyzing, interpreting, and generating human language.	Extracting structured information from unstructured clinical notes and EHRs; literature mining; decision support.	Converting unstructured EHR notes into analyzable data for predictive modeling of disease progression.	[34,43]
Radiomics	The high-throughput extraction of quantitative features from medical images to characterize tissue heterogeneity and disease phenotypes.	Predicting treatment response, prognosis, and correlating imaging features with genomic data.	Predicting PD-L1 expression, immunotherapy response, and overall survival from CT-based radiomic features.	[37,38]
RL	A paradigm where an algorithm learns optimal actions through trial-and-error interactions with an environment to maximize a cumulative reward.	Adaptive treatment planning, personalized dosing strategies, and diagnosis under uncertainty.	Optimizing radiotherapy dose fractions based on sequential CT scans during NSCLC therapy.	[39]
Generative AI	Models that learn the underlying distribution of data to generate new, synthetic data samples (e.g., Generative Adversarial Networks).	Data augmentation, synthetic image generation to overcome data scarcity, and predictive modeling.	Creating realistic synthetic medical images to train robust DL models when annotated datasets are limited.	[34]

**Table 4:** Critical comparison of AI strategies in lung cancer: limitations and context-specific solutions.

AI Strategy	Key Limitations	Proposed Context-Specific Solutions
Radiomics	Feature instability: Variations in scanners, acquisition protocols, and reconstruction algorithms. Low biological interpretability: Extracted features often lack clear biological meaning. Overfitting risk: High-dimensional feature sets relative to sample size. Single-modality limitation: Reflects morphology but not molecular or functional tumor biology.	Standardization: Adopt imaging biomarkers standardization initiatives (e.g., IBSI) and phantom-based calibration. Dynamic/delta-radiomics: Use longitudinal scans to track feature changes over time for better biological relevance. Integration with DL: Use DL for automated, stable feature extraction and reduction in handcrafted feature redundancy. Context: Best for non-invasive, low-cost screening, nodule characterization, and longitudinal monitoring where tissue is unavailable.
Genomics/molecular AI	Tumor heterogeneity: Single biopsy may not represent entire tumor genomic landscape. High cost and turnaround time for sequencing. Data sparsity: Many rare mutations with limited training examples. Functional interpretation challenge: Distinguishing driver from passenger mutations.	Liquid biopsy integration: Use ctDNA for real-time, systemic genomic profiling to capture heterogeneity. Pathway-centric models: Shift from single-gene to pathway/network-level analysis for improved biological insight. Transfer learning: Pre-train on large pan-cancer genomic datasets and fine-tune on smaller lung cancer cohorts. Context: Essential for targeted therapy selection, resistance mechanism analysis, and clinical trial matching in advanced/metastatic disease.
Multimodal AI	Data fusion complexity: Aligning and scaling heterogeneous data types (images, genomics, EHRs). Missing data: Incomplete multimodal datasets in real-world settings. Interpretability challenges: Increased model complexity reduces transparency. Computational and infrastructural demands.	Early vs. late fusion strategies: Use late fusion for modular, interpretable models; early fusion for capturing deep cross-modal interactions. Generative imputation: Use GANs or VAEs to generate plausible synthetic data for missing modalities. Attention mechanisms: Incorporate cross-modal attention to highlight contributing data sources and improve explainability. Context: Ideal for comprehensive patient profiling, early detection in high-risk cohorts, and personalized therapy where multiple data sources are available.
Deep learning (image-based)	‘Black box‘ nature: Limited explainability, especially in clinical high-stakes decisions. Large annotated datasets required: Expensive and time-consuming to curate. Generalizability issues: Performance drops on external datasets from different institutions. Computationally intensive training.	Explainable AI integration: Use SHAP, LIME, or attention maps to visualize decision regions. Federated learning: Train across institutions without sharing raw data to improve generalizability and data privacy. Weakly/semi-supervised learning: Reduce annotation burden by using slide-level labels or leveraging unlabeled data. Context: Most effective in high-volume imaging tasks (e.g., screening, digital pathology) where data quantity is sufficient and workflow automation is needed.
Liquid biopsy + AI	Low tumor DNA fraction in early-stage disease. Technical noise and artifacts from sequencing and bioinformatics pipelines. Lack of standardized bioinformatics pipelines for ctDNA analysis. Limited spatial information compared to tissue biopsy.	Multimodal enrichment: Combine with protein markers, fragmentomics, or methylation patterns to increase sensitivity. Longitudinal monitoring: Use AI to detect minimal residual disease and early recurrence through trend analysis. Standardized bioinformatics workflows: Adopt consensus pipelines (e.g., by FDA/CAP) for reproducibility. Context: Promising for early detection, monitoring treatment response, and detecting resistance mutations in advanced NSCLC.
Digital/computational pathology	Large file sizes and computational demands for whole-slide images. Inter-observer variability in ground truth annotations. Staining and scanning variability across labs. Limited clinical integration into pathology workflows.	Tile-based processing and cloud computing: Enable scalable analysis of WSIs without local hardware constraints. Consensus annotation platforms: Use multi-reader annotations with adjudication to improve label quality. Stain normalization algorithms: Standardize color and intensity across different scanners and protocols. Context: Optimal for tumor grading, subtyping, and biomarker quantification (e.g., PD-L1, TILs) in pathology labs with digital infrastructure.

**Table 5:** Data landscapes for AI in lung cancer.

Data Modality	Description and Role in AI	Key Insights	Integration Examples	Refs.
Medical imaging	Radiology data (e.g., CT, PET/CT) forms a cornerstone for diagnostic AI, providing morphological and structural information.	Enables early detection, nodule characterization, and extraction of radiomic features that correlate with tumor biology.	Automated RWD integration: Techniques bridge gaps between disparate data sources (hospital, academic, commercial) to enhance cancer outcome predictions.	[44]
Genomic data	Includes DNA sequencing, RNA expression profiles, and mutation data, offering a molecular characterization of the tumor.	Critical for understanding tumor heterogeneity, identifying actionable mutations, and tailoring targeted therapies.	Clinico-genomic databases: Real-world databases (e.g., Flatiron Health-Foundation Medicine) link genomic information with clinical data to characterize mutation-treatment effects. European perspective: Highlights the importance of real-world genomic data for precision oncology.	[45,46]
Electronic health records (EHRs)	A rich repository of patient-specific clinical information, including diagnostic reports, procedural notes, and unstructured clinical narratives.	Provides comprehensive clinical histories for predictive modeling of disease progression and uncovering patient trajectories.	NLP transformation: NLP techniques convert unstructured EHR notes into analyzable, structured data for AI models. Predictive modeling: AI-driven analysis of EHRs unveils patient trajectories and identifies potential biomarkers.	[47,48]
Real-world data (RWD)	Encompasses data collected outside of controlled clinical trials, including EHRs, claims data, and patient registries.	Reflects actual clinical practice and patient outcomes, providing insights into treatment effectiveness and disease heterogeneity.	Improved prognostics: DL algorithms trained on RWD show improved predictive capabilities for patient prognosis. Underutilization: Challenges in standardization and integration across sources limit full potential.	[43,48]
Integrated multi-modal data	The convergence of imaging, genomic, and clinical data to create a holistic view of the patient’s disease state.	Overcomes limitations of single data types, enabling more precise diagnostics, prognostication, and personalized interventions.	Deep patient models: Development of models like DeePaN, which integrates EHRs and genomic data to predict treatment responses. Foundation Models: Large-scale AI models trained on extensive, integrated multi-omics and clinical datasets to uncover complex biological mechanisms.	[50,51]

**Table 6:** Key challenges and proposed solutions for the clinical translation and validation of AI models in lung cancer care.

Challenge	Limitations	Proposed Solutions and Strategies	Refs.
Data and standardization	Lack of standardized data acquisition protocols; heterogeneity in data quality and formats limits algorithmic robustness and generalizability.	Develop unified norms for medical data collection; use federated learning (e.g., Federated Tumor Segmentation initiative); create large, diverse, multi-institutional datasets.	[198,213]
Algorithmic and technical limitations	Models struggle with subtle early-stage radiological features; difficulty distinguishing cancer types with shared biomarkers; lack of robustness across diverse datasets.	Develop more sophisticated architectures (e.g., multimodal DL); employ techniques like uncertainty quantification; rigorous multi-center validation.	[201,207]
Validation and reporting gaps	Insufficient external validation on independent, diverse cohorts; critical reporting standards (e.g., ethnicity, model calibration) are frequently overlooked.	Conduct large-scale, multi-center validation studies (e.g., PANDA challenge); adhere to rigorous reporting guidelines for transparency and reproducibility.	[209,214]
Clinical acceptance and workflow integration	Low levels of trust from clinicians; ‘black box’ problem; lack of seamless integration into existing clinical workflows and EHR systems.	Develop explainable AI and user-friendly interfaces; incorporate confidence metrics (e.g., uncertainty quantification); foster interdisciplinary collaboration.	[116,213]
Overarching strategy	The absence of a holistic, integrated approach from development to deployment.	Adopt a ‘vertically integrated’ approach that considers data lifecycles, impact evaluation, and production from the outset. Cultivate interdisciplinary talent.	[198,213]

**Table 7:** Methods for enhancing the interpretability and trustworthiness of AI models for lung cancer detection.

Aspect	Description and Function	Role in Clinical Translation	Refs.
Core techniques	SHAP: A game theory-based method that assigns each feature an importance value for a specific prediction. LIME: Approximates a complex model with a simpler, interpretable one locally around a specific prediction.	Provides post hoc explanations for individual AI decisions, making ‘black box’ models more transparent. Identifies key features (e.g., nodule size, texture) that drove a diagnosis.	[216,217]
Clinical utility and impact	Generates visual explanations (e.g., heatmaps, feature importance plots) that align with clinical reasoning. This bridges the gap between computational output and medical intuition.	Enhances clinician trust and confidence in AI systems by providing a clear, understandable rationale for recommendations, which is vital for adoption in high-stakes diagnostics.	[219,222]
Implementation and examples	Integrated into AI models like DeepXplainer for lung cancer detection. Used alongside AutoML to create systems that are both accurate and interpretable.	Facilitates a ‘second look’ by clinicians, allowing them to verify the AI’s logic and integrate its findings into their own diagnostic workflow more effectively.	[216,220]
Limitations and challenges	Explanations can be complex for non-experts; methods like SHAP can be computationally expensive; potential for misinterpretation of the generated explanations remains.	Ongoing research focuses on refining these methods to be more efficient and on creating more intuitive visualization tools tailored for clinical end-users.	[221,223]

**Table 8:** Summary of the primary ELSI and proposed mitigation strategies for AI integration in lung cancer care.

Domain	Key Challenges	Considerations and Mitigation Strategies	Refs.
Ethical and fairness	Algorithmic bias: AI models trained on non-representative data can perpetuate and amplify health disparities, leading to unfair outcomes for underrepresented groups.	Curate diverse, representative datasets; implement bias detection and mitigation strategies during model development; conduct fairness audits across demographic subgroups.	[224,227,228]
Legal and liability	Accountability and liability: Unclear legal responsibility for diagnostic errors or patient harm caused by AI recommendations. Issues of data confidentiality and informed consent for AI use.	Establish clear legal frameworks defining responsibilities of developers, providers, and users; update informed consent processes to include AI; develop standards for safety and accountability.	[225,226,231]
Social and equity	Equity of access: Risk that AI tools could worsen existing health disparities if deployed primarily in well-resourced settings. Societal trust: Public and clinician skepticism due to AI’s opacity and potential for error.	Develop policies that promote equitable access to AI-driven care; foster public and clinician engagement through transparency and education; adopt gender-sensitive and culturally aware approaches.	[226,229,230]
Governance and trustworthiness	Lack of robust governance: Absence of comprehensive frameworks for ensuring AI systems are transparent, accountable, and aligned with ethical principles like autonomy, beneficence, and justice.	Implement multidisciplinary ethical governance; use auditing as a tool for ongoing evaluation (e.g., 3-layered audits); embed ethical principles into the entire AI lifecycle from design to deployment.	[227,228,232]

**Table 9:** Economic value drivers and assessment challenges of integrating AI into lung cancer diagnosis, treatment, and development.

Application	Economic Impact and Cost-Drivers	Key Findings	Refs.
Screening and diagnosis	Cost savings: Reduces false positives and unnecessary follow-up procedures (e.g., biopsies). Efficiency: Streamlines diagnostic workflows, reducing delays and resource use.	AI as a second reader in low-dose CT screening can improve cost-effectiveness by minimizing false positives. AI models streamline diagnosis, enabling faster, more accurate early detection.	[235,236,237]
Treatment personalization	Therapeutic efficiency: Aids in selecting effective therapies, avoiding costs of ineffective treatments and managing adverse events. Optimization: Personalizes therapy, improving outcomes and resource allocation.	AI can personalize therapy for complex cases like NSCLC, potentially avoiding costly, ineffective treatments. AI-enhanced radiomics in PET/CT improves tumor characterization for precise treatment selection.	[85,238,239]
Drug development	Accelerated pipelines: Reduces time and cost of drug discovery by identifying targets and candidates more efficiently. Resource optimization: Improves success rates in preclinical and clinical stages.	AI meets the demand for faster, cost-effective drug discovery by predicting drug-target interactions and generating novel chemical entities.	[244,245]
Operational efficiency and access	Hospital workflows: Optimizes resource allocation, reduces hospital stays, and streamlines decision-making. Accessibility: AI-enabled mobile units can improve access in underserved populations.	AI improves operational efficiency in hospital settings. Deployment of mobile low-dose CT units with AI can enhance accessibility and cost-effectiveness in rural/underserved areas.	[245,247]
Methodological challenges	Assessment complexity: Current health economic models may be inadequate for evaluating AI’s unique features (e.g., continuous learning, algorithm updates). Evidence gaps: Lack of long-term cost–benefit analyses and data on patient-reported outcomes.	Health technology assessment frameworks need adaptation for AI-based devices. Economic evidence for complex AI interventions, like liquid biopsy, remains limited and requires sophisticated modeling.	[238,240,241]

## Data Availability

No new data were created or analyzed in this study.

## Abbreviations

The following abbreviations are used in this manuscript:

AI	Artificial intelligence
CNNs	Convolutional neural networks
CT	Computed tomography
DeePaN	Deep patient graph convolutional networks
DL	Deep learning
EGFR	Epidermal growth factor receptor
EHRs	Electronic health records
ELSI	Ethical, legal, and social implications
HDI	Human development index
LIME	Local interpretable model-agnostic explanations
ML	Machine learning
NLP	Natural language processing
NSCLC	Non-small cell lung cancer
PD-L1	Programmed death ligand 1
PET	Positron emission tomography
RL	Reinforcement learning
RWD	Real-world data
SHAP	SHapley Additive exPlanations
WSIs	Whole-slide images
